# High-THC *Cannabis* Smoke Impairs Incidental Memory Capacity in Spontaneous Tests of Novelty Preference for Objects and Odors in Male Rats

**DOI:** 10.1523/ENEURO.0115-23.2023

**Published:** 2023-12-06

**Authors:** Ilne L. Barnard, Timothy J. Onofrychuk, Aaron D. Toderash, Vyom N. Patel, Aiden E. Glass, Jesse C. Adrian, Robert B. Laprairie, John G. Howland

**Affiliations:** 1 Department of Anatomy, Physiology, and Pharmacology, University of Saskatchewan, Saskatoon, Saskatchewan, S7N5E5, Canada; 2 College of Pharmacy and Nutrition, University of Saskatchewan, Saskatoon, Saskatchewan, S7N5E5, Canada; 3 Department of Pharmacology, College of Medicine, Dalhousie University, Halifax, Nova Scotia, B3H 4R2, Canada; 4 Department of Computer Science, University of Saskatchewan, Saskatoon, Saskatchewan, S7N5C9, Canada

**Keywords:** cannabinoid, machine learning, recognition memory

## Abstract

Working memory is an executive function that orchestrates the use of limited amounts of information, referred to as working memory capacity, in cognitive functions. *Cannabis* exposure impairs working memory in humans; however, it is unclear whether *Cannabis* facilitates or impairs rodent working memory and working memory capacity. The conflicting literature in rodent models may be at least partly because of the use of drug exposure paradigms that do not closely mirror patterns of human *Cannabis* use. Here, we used an incidental memory capacity paradigm where a novelty preference is assessed after a short delay in spontaneous recognition-based tests. Either object or odor-based stimuli were used in test variations with sets of identical [identical stimuli test (IST)] and different [different stimuli test (DST)] stimuli (three or six) for low-memory and high-memory loads, respectively. Additionally, we developed a human-machine hybrid behavioral quantification approach which supplements stopwatch-based scoring with supervised machine learning-based classification. After validating the spontaneous IST and DST in male rats, 6-item test versions with the hybrid quantification method were used to evaluate the impact of acute exposure to high-Δ^9^-tetrahydrocannabinol (THC) or high-CBD *Cannabis* smoke on novelty preference. Under control conditions, male rats showed novelty preference in all test variations. We found that high-THC, but not high-CBD, *Cannabis* smoke exposure impaired novelty preference for objects under a high-memory load. Odor-based recognition deficits were seen under both low-memory and high-memory loads only following high-THC smoke exposure. Ultimately, these data show that *Cannabis* smoke exposure impacts incidental memory capacity of male rats in a memory load-dependent, and stimuli-specific manner.

## Significance Statement

Incidental memory refers to the limited amount of information encoded by chance during behavior. How psychoactive drug exposure affects incidental memory is poorly understood, particularly for *Cannabis* exposure. To address this question, we validated object-based and odor-based spontaneous incidental memory tests in male rats using a novel human-machine hybrid scoring method. Using these tests, we show exposure to high-Δ^9^-tetrahydrocannabinol (THC), but not high-CBD, *Cannabis* smoke impairs incidental memory under high-memory loads in object-based tests and both high-memory and low-memory loads in the odor-based tests. Our results highlight cannabinoid-specific effects on incidental memory in male rats using a validated *Cannabis* smoke exposure method, which have broad implications for the impacts of human use of *Cannabis* on cognition.

## Introduction

Working memory is an executive function that orchestrates the use of limited amounts of information in cognitive functions like learning and memory (D’Esposito et al., 1995; Wilhelm et al., 2013; Eriksson et al., 2015; Constantinidis and Klingberg, 2016). In humans, Δ^9^-tetrahydrocannabinol (THC), the main psychoactive constituent of *Cannabis*, impairs working memory following both acute and chronic *Cannabis* exposure, likely by action at the cannabinoid type 1 receptor (Curran et al., 2002; Ilan et al., 2004; Bossong et al., 2012; D’Souza et al., 2012; Crane et al., 2013; Cousijn et al., 2014; Ligresti et al., 2016; Owens et al., 2019; Adam et al., 2020). The working memory impairments produced by *Cannabis* have been interpreted as resulting from disruptions of the active maintenance, limited capacity, interference control, and flexible updating subconstructs of working memory (Barch and Smith, 2008). In contrast, studies in rodents demonstrate both THC-mediated impairments and improvements in working memory function (Varvel et al., 2001; de Melo et al., 2005; Goonawardena et al., 2010; Bruijnzeel et al., 2016; Blaes et al., 2019; Barnard et al., 2022). These inconsistent findings may be attributable to differences in the behavioral tasks used, cannabinoid dosage, exposure timelines, and routes of administration (Klausner and Dingell, 1971; Nguyen et al., 2016; Hložek et al., 2017; Baglot et al., 2021; Wiley et al., 2021). Importantly, previous rodent studies have not directly assessed the effects of *Cannabis* exposure on working memory capacity. Working memory capacity is essential for higher cognitive operations critical to everyday function and can be impaired in disorders like schizophrenia and Parkinson’s disease (Goldman-Rakic, 1999; Piskulic et al., 2007; Gold et al., 2019).

A shortcoming in rodent literature is that traditional rodent working memory capacity tests mimic n-back or recall working memory tests used in humans and require a long training period, learned rules, and considerable experimental involvement (Kirchner, 1958; Daneman and Carpenter, 1980; Dudchenko, 2004; Cowan, 2010; Dudchenko et al., 2013; Oomen et al., 2013; Wilhelm et al., 2013; Vorhees and Williams, 2014; Scott et al., 2020; Barnard et al., 2022). Spontaneous recognition tests circumvent these weaknesses by relying on rodents’ innate novelty seeking behavior as shown by preferential interaction with a novel stimulus after a delay (Ennaceur and Delacour, 1988; Ennaceur and Aggleton, 1994; Broadbent et al., 2004; Sannino et al., 2012). These tests measure incidental memory capacity, which is the limited amount of information that is encoded by chance during spontaneous exploration. It is noteworthy that incidental memory capacity differs from working memory capacity, as information is encoded without the intent for future use. Novelty preference can be used to assess incidental memory capacity in mice under low-memory and high-memory loads through the Identical and Different Objects Tasks, respectively (Sannino et al., 2012; Olivito et al., 2016, 2019; Torromino et al., 2022). Therefore, the first goal of the present study was to validate these tests in male rats using the identical stimuli test (IST) and different stimuli test (DST) with objects. Our second goal was to develop and validate olfactory versions of these tests to evaluate incidental memory for odors. We chose to perform this initial validation with male rats given the recently reported sex differences in the neural circuitry underlying performance of the tests with objects in mice (Torromino et al., 2022).

For all test variations, novelty preference was inferred by measuring the relative amount of interaction behavior exhibited at novel and previously experienced stimuli after a short delay. Typical approaches to quantifying rodent behavior for spontaneous interaction tests are generally laborious, prone to human subjectivity, and lack objective analysis steps that can be verified and reproduced (Anderson and Perona, 2014). Recent advances in automated behavioral analysis have enabled researchers to obtain a detailed and objective record of a diversity of complex behaviors across species (Nilsson et al., 2020; Cui et al., 2021; C. Winters et al., 2022; Newton et al., 2023; Slivicki et al., 2023). Here, we automatically quantified interaction events using a supervised machine learning-based analysis approach with DeepLabCut (Mathis et al., 2018) and simple behavioral analysis (SimBA; Nilsson et al., 2020), then on manual inspection of supervised machine learning predictions, suboptimal predictions were supplemented by human stopwatch scoring to form a human-machine hybrid scoring method. By automatically predicting interaction events frame-by-frame, several secondary behavioral measures, including approach latency and interaction bout count, were easily calculated and provide a more complete characterization of novelty preference to infer incidental memory capacity. To our knowledge, the present study is the first demonstration of supervised machine learning-based behavioral analysis in the context of a spontaneous interaction-based test.

Using validated spontaneous tests and the hybrid scoring method, our second goal was to assess the effects of *Cannabis* smoke exposure on novelty preference to infer incidental memory capacity. We tested male rats shortly after acute exposure to the smoke of either high-THC or high-CBD-containing *Cannabis* buds using an exposure paradigm validated with rats (Barnard et al., 2022; Roebuck et al., 2022). We found that high-THC, but not high-CBD, smoke impaired performance of male rats in the tests in a stimuli-specific manner.

## Materials and Methods

### Subjects

Adult (two to four months of age) male Long–Evans rats (*n* = 92; Charles River Laboratories) were pair housed in a vivarium in standard ventilated cages with *ad libitum* water and food, and a plastic tube for environmental enrichment on a 12/12 h light/dark cycle (starting at 7 A.M.). For establishment and validation of IST and DST with objects and odors, 52 rats were used; 48 additional rats were used to evaluate the impact of acute *Cannabis* smoke exposure on novelty preference. Rats were tested at the same time of day between the hours of 7:30 A.M. and 6 P.M. All procedures followed guidelines from the Canadian Council on Animal Care and were approved by the University of Saskatchewan Animal Research Ethics Board.

### Apparatus and testing materials

**Figure 1.**
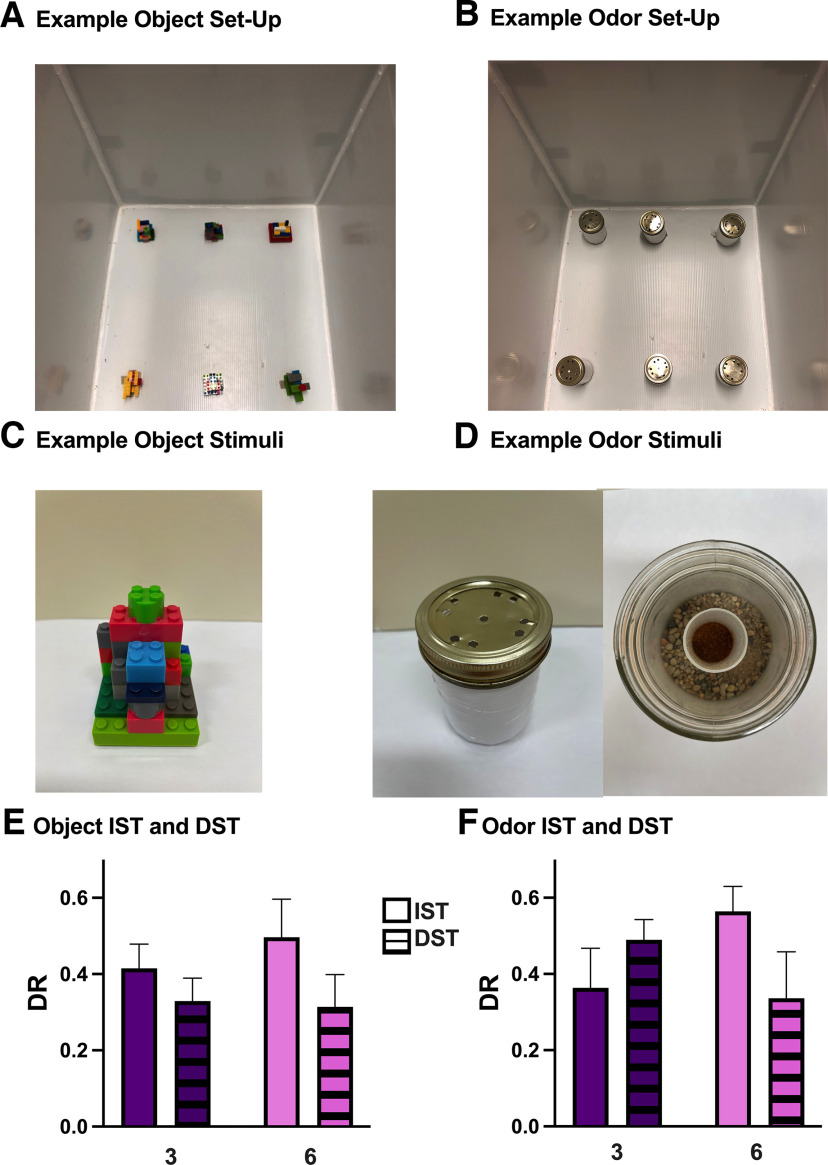
The validation and establishment of the IST and DST with objects and odors. ***A***, A picture of an example object set-up is shown. Objects are displayed in six positions in a white-corrugated plastic box. ***B***, A picture of an example odor set-up is shown. Odors are displayed in six positions in a white-corrugated plastic box. ***C***, An example of an object stimuli. ***D***, Example of an odor stimuli. ***E***, Object interaction was measured using DRs to evaluate novelty preference using 3-objects and 6-objects. Male rats explore the novel object significantly more than the familiar objects in the IST and DST with both 3-objects and 6-objects. No differences in novelty preference or exploration times are seen between the IST and DST, or between 3-object and 6-object versions. ***F***, Odor interaction was also measured using DRs to evaluate novelty preference using 3-odors and 6-odors. Male rats explore the novel odor significantly more than the familiar odors in the IST and DST with both 3-odor and 6-odor. No differences in novelty preference or exploration times are seen between the IST and DST, or between the 3-odor and 6-odor versions. Data are represented as mean ± SEM.

Rats were handled in the testing room (3 min/d for 3 d) and subsequently habituated to both the testing apparatus (10 min for 2 d) and to the smoke chamber apparatus (20 min for 2 d). Rats were tested in a white corrugated plastic box (60 × 60 × 60 cm) with the stimuli evenly presented between two opposing walls at three positions (Fig. 1; 9 cm from side of box, 21.5 cm apart from each other). Object stimuli were created from a variety of LEGO pieces of different sizes and colors with an average size of 7 × 10 cm. LEGO was chosen to maintain consistency between different object sets. Odor stimuli were created using 250-ml glass canning jars. The jars were filled with sand for stability, and to provide a resting place for a small plastic vile filled half-way with a powered spice (lemon pepper, dill, sage, onion, anise, cloves, ginger, cumin, cocoa, celery salt, coffee, cinnamon, garlic, or oregano). Holes were drilled in the lids of the jars to allow the rats to smell the spices. All items were affixed to the testing apparatus with Velcro at one of six positions to prevent them from being displaced during the test.

### Spontaneous incidental memory test protocol

**Figure 2.**
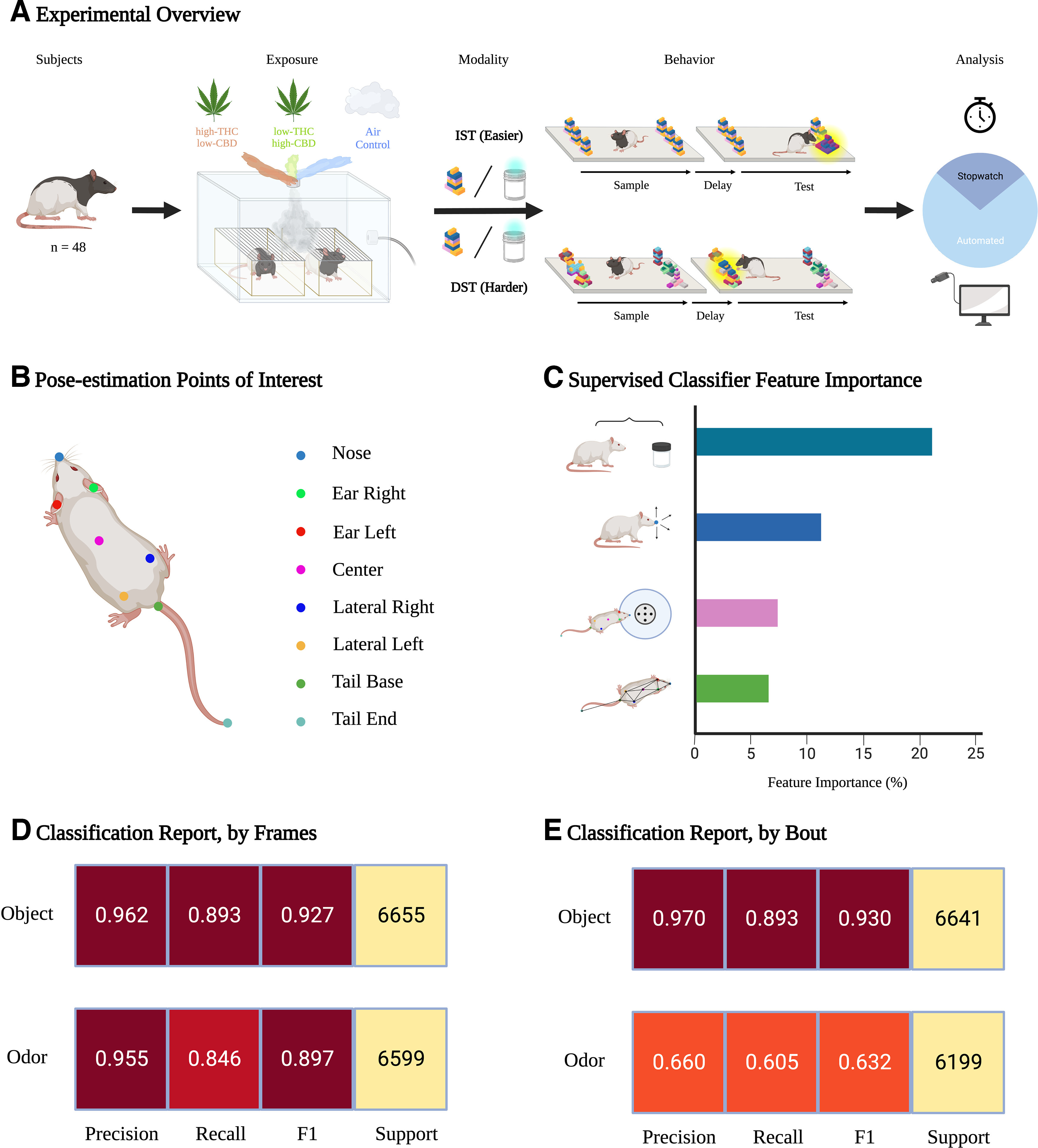
Experimental overview for acute *Cannabis* exposure and behavioral classifier training. ***A***, Schematic representation of the experimental design. Male Long–Evans rats (*n* = 48) were used for this study. Using a repeated measures experimental design, each rat was exposed to high-THC *Cannabis* smoke, low-THC *Cannabis* smoke, and an Air Control condition. Male rats were exposed 20 min before the start of behavioral testing. Each male rat either underwent the 6-object IST and 6-object DST, or the 6-odor IST and 6-odor DST. The order in which the IST and DST were performed was randomized. Rat behavior was quantified using traditional stopwatch scoring and by automated supervised machine learning-based behavioral analysis. Suboptimal supervised machine learning predictions were replaced by stopwatch scoring, constituting a hybrid scoring approach. ***B***, Illustration of the point-of-interest configuration used for pose-estimation analysis. We chose the number and position of points in accordance with the SimBA eight-point configuration. SimBA requires a standardized and specific position (and number) of points. Users should decide what SimBA configuration will be used (single animal, multianimal, point number) before network training with DeepLabCut. ***C***, Visualization of the relative feature importance of the four features clusters. In short, the 40 most important features were systematically categorized into distinct clusters, then we summed the feature importance’s of individual features within each cluster. The raw features importance log is included under “assessment + logs” for each classifier within our GitHub repository. ***D***, Classifier performance metrics for the object (top) and odor (bottom) models. Test frames were randomly extracted from the dataset (20% test, 80% train). ***E***, Classifier performance metrics for the object (top) and odor (bottom) models. Test bouts were randomly extracted from the dataset (20% test, 80% train). See Extended Data Figures 2-1, 2-2, 2-3, and 2-4 for more information regarding the supervised machine learning approach and validation. This figure was created using BioRender.

To validate the IST and DST with objects, 24 naive rats performed both the 3-object and 6-object variations (Fig. 1). Twenty naive rats were used to establish the 3-odor and 6-odor IST and DST. Using a within-subjects design, 48 additional rats performed both the IST and DST with objects and odors 20 min after *Cannabis* smoke exposure (Fig. 2*A*). The order of tests was quasi-counterbalanced, and rats had a 2-d washout period between tests. On the test day, the testing box was prepared with two sets of six stimuli for the test and paradigm being performed (Figs. 2*A*, 4*A*,*B*, 5*A*,*B*). The rat was then placed into the testing box for the sample phase, for a duration of 5 min. Following the sample phase, the rat was taken out of the testing box and placed inside a transport cage for 1 min. During the delay, all stimuli were replaced for the test phase. Then, the rat was placed back into the box for the test phase (5 min). The testing box and the stimuli were cleaned with 70% ethanol after each phase.

10.1523/ENEURO.0115-23.2023.f2-1 Extended Data Figure 2-1 Mean tracking confidence for each point-of-interest, by video. To calculate the mean tracking confidence for each video, the average of the likelihood column associated with each point of interest was calculated. Download Figure 2-1, TIF file.

10.1523/ENEURO.0115-23.2023.f2-2 Extended Data Figure 2-2 Model hyperparameters used for classifier training. A metadata csv file is included under “assessment + logs” for each classifier within our GitHub repository. Previous studies have shown that creating a balanced dataset by using the model hyperparameters of “random under sampling” or “random over sampling” lead to better classifier performance; however, we found that using these features dramatically decreased classifier performance and lead to equal classifier predictions across the data frame. Therefore, we chose to not use these hyperparameters for analysis, and accounted for the unbalanced dataset by setting a relatively low discrimination threshold. For both classifiers, a discrimination threshold of 0.35 and a minimum bout duration of 50 ms was used (Extended Data Fig. 2-3). Download Figure 2-2, TIF file.

**Figure 3.**
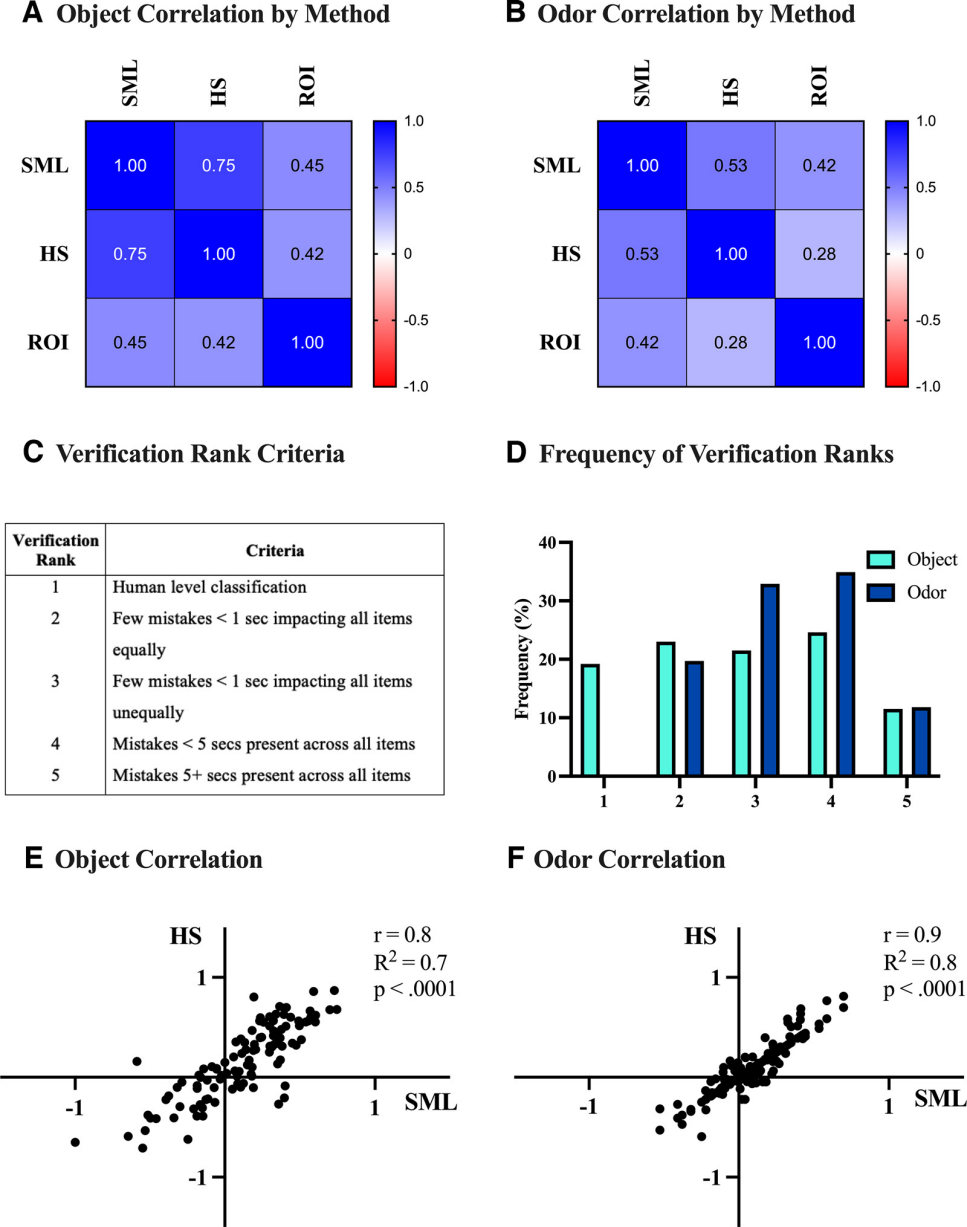
Comparison between human stopwatch and supervised machine-learning generated output. ***A***, Correlation matrix between methods of quantifying rat-object interaction. This comparison was made between supervised machine-learning (SML), human-stopwatch (HS), and region-of-interest (ROI)-generated interaction times. Interaction times by object was quantified using each scoring method, then the correlation between interaction DRs was assessed. ***B***, Correlation matrix between methods of quantifying rat-odor interaction. Interaction times by odor was quantified using each scoring method, then the correlation between interaction DRs was assessed. ***C***, Criteria used to rank automated classification. Each video was manually viewed for accurate classification, where a verification rank was assigned based on objective criteria. ***D***, Frequency of verification rank assignment by type of stimuli. Videos with a verification rank less than three were excluded from final analysis and replaced by human stopwatch scoring. Approximately 80% of object videos and 60% of odor videos met inclusion criteria, respectively. ***E***, Correlation between human stopwatch and SML-generated DRs on object videos meeting inclusion criteria, indicating a moderate-to-high correlation (*r*_(109)_ = 0.83, *p* < 0.0001). ***F***, Correlation between human stopwatch and SML-generated DRs on odor videos meeting inclusion criteria, indicating a moderate-to-high correlation (*r*_(77)_ = 0.87, *p* < 0.0001). See Extended Data Figures 3-1 and 3-2 for additional information regarding the scoring and the ranking of videos by *Cannabis* treatment.

10.1523/ENEURO.0115-23.2023.f2-3 Extended Data Figure 2-3 Representative plot of classifier predictions across a complete video (9000 frames, 5-min video). We chose a discrimination threshold of 0.35 as it corresponds to the middle segment of obvious probability spikes and excludes the majority of noise below 0.2. We assessed model performance in two ways, both of which are integrated in the SimBA GUI (Extended Data Fig. 2-2). First, we generated performance metrics (precision, recall, F1) by randomly splitting the aggregate training set (all human-annotated frames from all videos within the project) into 80% training frames and 20% test frames. Said differently, for a given behavioral video, a fraction of interaction-containing frames was used for model training, then a smaller fraction of frames was used for testing whether the model can accurately predict whether rat-stimulus interaction occurs in each test frame. As shown below, we found that both the object and odor classifiers generated excellent performance metrics when assessed in this manner. However, a fundamental problem with this assessment method is that for a given interaction bout, there may be both test and training frames, so the model is predicting interaction between two known sub-bouts of interaction (visualized- 1 = known interaction, test = test frame that the model must make a prediction on: 1-1-1-1-1-test-1-1-1-1). Therefore, to assess performance without the confound of intrabout test frames, we segregated the aggregate training into interaction bouts, then split the segregated training set into 80% training bouts and 20% test bouts. We found that the performance of the object classifier changed marginally with this change, but performance metrics for the odor classifier significantly decreased when assessed in this manner. While we content that assessing classifier performance by-bout is a more conservative and representative method, an important caveat is that classifier performance on a completely model-naive video is not assessed by either of these methods. This is important to consider because researchers will typically implement this analysis method to automatically quantify behavior for a large dataset, where only a fraction of this dataset is used for training. We did not include a by-video classifier analysis as this is not integrated into SimBA, but we contend that future research and software development should implement this performance assessment method to capture the accuracy of classifier predictions most accurately on model naive behavioral videos. Download Figure 2-3, TIF file.

**Figure 4.**
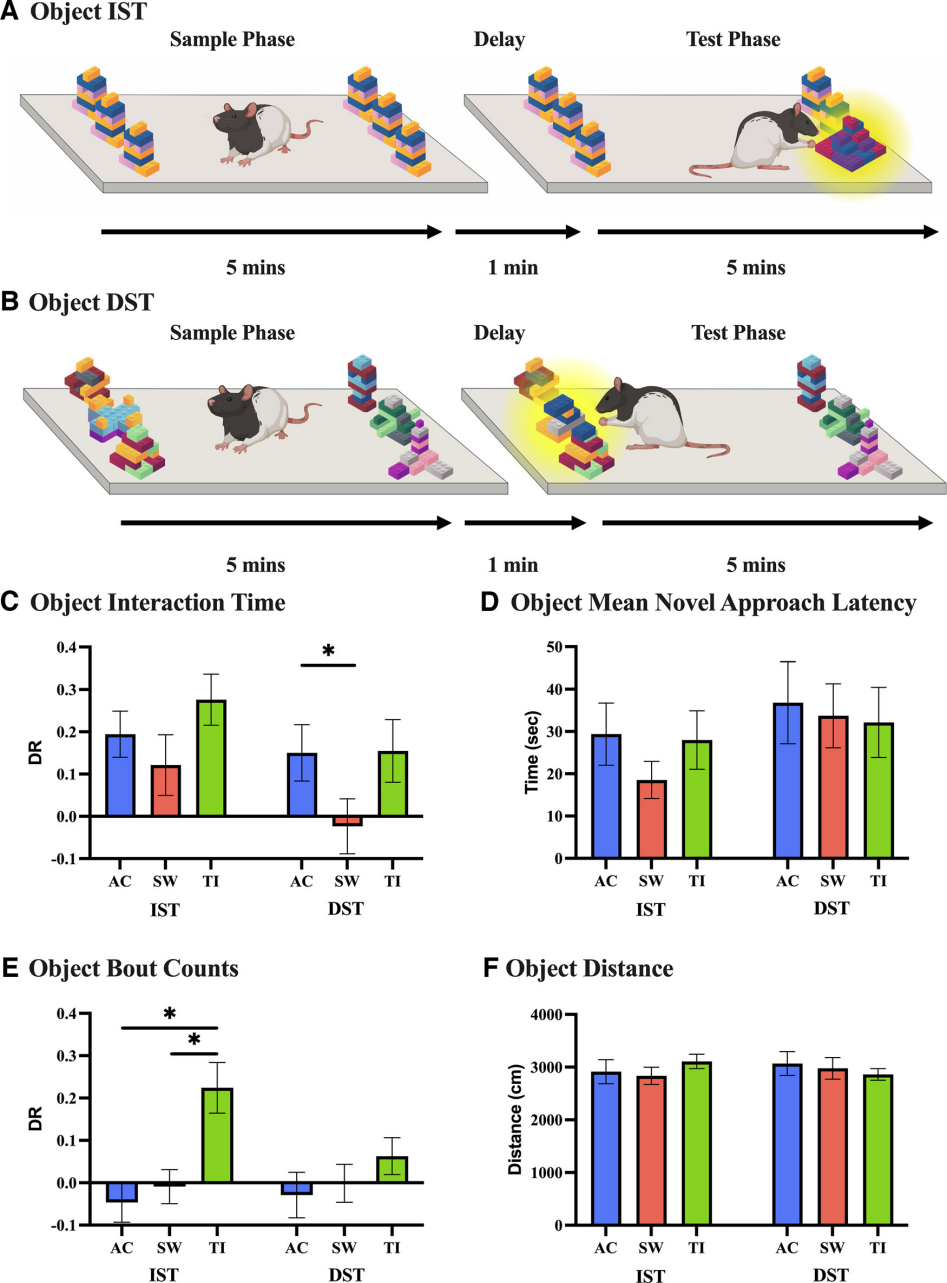
High-THC *Cannabis* smoke exposure impacts novelty preference under high-memory (DST) loads using object stimuli, with no impact on distance traveled, frequency of item visitation, or approach latencies. ***A***, An example IST with objects is visualized, showing six identical objects in the sample phase, with a novel object introduced after a 1-min delay in the test phase. ***B***, A DST with objects variation is shown, with an identical test progression, but instead starts with six different objects in the sample phase. ***C***, Interaction measured as time spent with an object was generated using the human-machine hybrid scoring approach and visualized using a discrimination ratio for both variations using object stimuli. No difference in treatment groups is seen in the 6-object IST (*n* = 64). In the 6-object DST (*n* = 66), a significant decrease in novelty preference is seen in the SW group in contrast to the AC group (*p* = 0.04). ***D***, The mean novel approach latency in the 6-object IST (*n* = 72) and 6-object DST (*n* = 69) variations is shown to be consistent between treatment groups. ***E***, To illustrate the frequency of visitations to the novel object in comparison to the familiar objects, bout counts are visualized using a discrimination ratio. A preference for novel visitations is seen in the 6-object IST (*n* = 65) AC and SW groups, with no difference in item visitations in the 6-object DST (*n* = 66). ***F***, The distance traveled (cm) in the 6-object IST (*n* = 72) and 6-object DST (*n* = 69) variations is comparable across treatment groups. Data represents mean ± SEM, *p < 0.05. Abbreviations: high-THC *Cannabis* smoke (SW); high-CBD *Cannabis* smoke (TI); Air Control (AC). This figure was created using BioRender.

10.1523/ENEURO.0115-23.2023.f2-4 Extended Data Figure 2-4 Precision recall curve visualizing changes in precision, recall, and F1 with classifier training. Raw data is included under “assessment + logs” for each classifier within our GitHub repository. Recall, precision, and by extension the F1 score are calculated from the entries of a confusion matrix. A confusion matrix tells us, given a set of observations belonging to at least two different classes and a classifier that attempts to label each, how many and what type of errors were made. The diagonal of the confusion matrix is the correct observations, the off diagonal are the errors. For a binary classifier, we are generally focused on one class over the other, thus the metrics we derive are chosen to represent how we did for the most important class. In our case “interaction” is the class we care about. In quantifying how our classifier for “interaction” did, we calculate the recall and precision. Recall is the proportion of all the possible “interaction” observations that our classifier predicted correctly. That is, the number of true positives (TP) divided by the total number of “interaction” observations (note the maximum number of true positives is all the “interaction” observations, in which case the recall equals 1, so a classifier that always predicts interaction will have perfect recall). Now there are many other metrics that could be computed, but the next most natural is the precision. Precision is the proportion of predicted “interaction” observations that were actual “interactions.” Or mathematically, the number of true positives divided by the total number of times our classifier predicted “interaction” (note it is not so easy to get perfect precision). Now we have two perfectly good numbers that quantify how our classifier did, the proportion of overall “interactions” that were recovered (recall) and the proportion of times our classifier predicted “interaction” and was correct (precision). It is not clear which is more important, so we combined the two as the F1 score as the harmonic mean of recall and precision. Why harmonic mean? We want an average of some kind, and the harmonic mean is the smallest of the 3 Pythagorean means (arithmetic mean, geometric mean, and harmonic mean). So, to have a high F1 score you must have high precision and recall, either one will drag the F1 score down nonlinearly. Download Figure 2-4, TIF file.

### *Cannabis* bud preparation and acute smoke exposure protocol

A high-THC (19.51%) and low-CBD (<0.07%) strain, Skywalker (Aphria Inc., lot #6216), and a high-CBD (12.98%) and low-THC (0.67%) strain, Treasure Island (Aphria Inc., lot #6812), were used for *Cannabis* smoke exposure as previously established (Barnard et al., 2022; Roebuck et al., 2022). All *Cannabis* was stored in light-protected containers at room temperature. On the day of testing, whole *Cannabis* buds were ground in a standard coffee grinder for 5 s. Then, 300 mg of the ground bud was measured and loaded into a ceramic coil that was part of a four-chamber inhalation system from La Jolla Alcohol Research. Rats were then loaded individually into small plastic cages and placed in the airtight Plexiglas chambers. A *Cannabis* combustion session started with a 5-min acclimation period, then a 1 min combustion occurred through three 5-s ignitions with a 15-s delay in-between each ignition. The temperature was set to 149°C, with a wattage of 60.1 W on the SV250 mod box. The smoke was then drawn into the clear Plexiglas chambers at a flow rate of 10–12 l/min. Following the 1 min combustion cycle, pumps were turned off for 1 min before they were turned back on for 13 min to gradually evacuate the smoke. Thus, the total exposure time was 15 min following initial ignition of the *Cannabis.* Rats were then moved to the testing apparatus to start the behavioral tests 20 min after the start of the combustion cycle. Boli left by the rats in the small plastic cages that housed them during combustion were then counted by an experimenter.

### Behavioral analysis

For validation of spontaneous incidental memory tests, behavioral videos were collected from an overhead perspective in black and white at a frame rate of 30 frames per second (fps) with a resolution of 720 × 480 pixels (Panasonic WV-BP334 1/3” B&W). Collected videos were manually scored using a conventional stopwatch method, where the duration of interaction at each stimulus was recorded.

To allow for automated behavioral analysis, behavioral videos for the *Cannabis* exposure experiment were recorded from an overhead perspective in full color at a frame rate of 30 fps and a resolution of 1080 × 1080 pixels (Logitech Brio 505, Logitech). To further standardize behavioral videos, we used the “batch preprocessing” module within SimBA to crop videos to only include the apparatus, to ensure standardized resolution and frame rate, and to the trim video length to desired experimental phases. Additionally, we chose to film all videos in a .mp4 format as this format is generally compatible with open-source video analysis software. More details regarding this process, and the subsequent steps in our supervised maching learning pipeline can be found here (https://github.com/HowlandLab/ILBTJO_NODB_SimBA_2023).

After filming, DeepLabCut (2.2.3) was used to continuously track the spatial location of eight user defined points-of-interest (Fig. 2*B*; Nath et al., 2019). Mean tracking confidence for each point-of-interest is shown in Extended Data Figure 2-1. To train the DeepLabCut model, we randomly extracted 300 frames from 60 representative behavioral videos, with an equal representation of the IST/DST and object/odor stimuli. Next, each frame was manually annotated, where a human annotator placed digital points-of-interest on the rat (Fig. 2*B*). Manually annotated frames were used to train a deep neural network-based model to predict the spatial location of points of interest for each frame across new videos. Nath et al. (2019) describe the procedure used in the present experiments for model training and subsequent video analysis using DeepLabCut. A pretrained ResNet-50 convolutional neural network (CNN) was then trained on 95% of annotated frames for 200,000 iterations, where 5% of frames were reserved for model assessment. After training, we analyzed the CNN learning curve to select an optimal model that performs well on both test and train data. Pose-estimation data were extracted from videos using a model trained for 80,000 iterations, which represents the iteration where test error is minimized, and the training error is saturated. Our model produced a training error of 4.89 and a test error of 4.35 using the default hyperparameters, without a p-cutoff filter applied. Finally, pose-estimation tracking files were filtered using the DeepLabCut native median filter model. It is important to note that annotated training frames for this experiment were added to an existing DLC project (training set = ∼1000 annotated frames). As the CNN was pretrained to predict the spatial position of key points, and all videos were filmed within an identical experimental apparatus, the number of additional required annotated frames to acquire high-fidelity pose-estimation data for the present experiment was likely lower than if the CNN was trained from scratch. The DLC model file used for analysis is freely available on GitHub (https://github.com/HowlandLab/ILBTJO_NODB_SimBA_2023), and any additional training data will be freely supplied on request.

We then trained a supervised machine learning-based behavioral classifier to predict interaction events based on movement features extracted from pose-estimation data (Goodwin et al., 2022). Nilsson et al. (2020) describe the detailed procedure used in the present experiments for model training and subsequent video analysis using SimBA. Classifier training was completed using the eight-point classical tracking version of the SimBA pipeline (SimBA-UW-tf-dev = 1.32.2). We trained two classifiers, one for object-based stimuli and one for odor-based stimuli, to predict interaction events across test variation. For each classifier, the training dataset consisted of user-annotated frames from ∼30 5-min videos, where each frame was assigned a binary label of “interaction” or “noninteraction.” The object-based and odor-based classifiers were trained on 28,586 and 32,872 frames of target “interaction” behavior, respectively. Before manual annotation, trimmed videos and filtered pose-estimation data were imported, then a scale factor was used to normalize variable camera filming heights to a known metric distance (experimental apparatus, dimensions = 60 × 60 cm). Additionally, each stimuli position was assigned a region-of-interest that was centered at each Velcro stimuli attachment point, with a defined radius extending ∼2 cm beyond the edge of stimuli. In total, 273 features were extracted from tracking data, where 251 features capture spatiotemporal relationships between points-of-interest, and 12 features capture region-of-interest (ROI)-related movement. We slightly deviated from the standard SimBA feature engineering approach by removing ROI-related features called “zone_cumulative_percent” and “zone_cumulative_time.” These features increase the prediction probability of a true class based on animal’s preferentially spending time in a defined ROI. While these features may be useful for predicting behaviors that only include in specific regions (e.g., rat dams retrieving pups from a nest), inclusion of these features in our project would bias predictions unequally between the six stimuli positions. For both the object and odor classifiers, the behavioral features most heavily weighted for model predictions include distance to stimuli, nose movements, region-of-interest, and spatial dynamics between points-of-interest (Fig. 2*C*). Feature importance clusters were created by extracting the 40 most important features from SimBA, then splitting features based on the following criteria: (1) features related to the distance to stimuli “distance to stimuli”; (2) features related to nose movements (e.g., Nose_movement_M1_sum_6) were clustered to “nose movements”; (3) features related to a subjects’ nose key point being located within a defined ROI surrounding stimuli were clustered to “region-of-interest”; (4) remaining features were clustered to a common “spatial dynamics between points-of-interest.” For the object classifier, we defined “interaction” as frames where the rat’s nose was within 2 cm of the object, while looking at and/or chewing the stimuli for a duration >50 ms. For the odor classifier, “interaction” was defined as frames where the rat’s nose was within 2 cm of the top of the odor jar, while looking at and/or chewing the stimuli for a duration >50 ms. Classifiers were built using the following hyperparameter set: n_estimators = 200, RF_criterion = entropy, RF_max_features = sqrt, RF_min_sample leaf = 2 (Extended Data Figs. 2-2, 2-3, 2-4). Precision, recall, and F1 scores for the classifiers are shown in Figure 2*D*,*E* and further described in the Extended Data. To account for instances of suboptimal supervised machine learning prediction, we created a five-tiered verification rank system, where supervised machine learning-generated predictions on videos with ranks of four or five were replaced by human stopwatch scoring for the final analysis (Fig. 3*C*,*D*).

10.1523/ENEURO.0115-23.2023.f3-1 Extended Data Figure 3-1 Inter-rater variability analysis between human scorers of varying experience levels. In short, 20 behavioral videos (counterbalanced for IST/DST and objects/odors) were scored for rat-stimulus interaction by three independent scorers of differing experience levels (master, experienced, beginner). We found a strong correlation between scorers of all experience levels, but a comparatively weaker correlation between experienced and beginner scorers. Download Figure 3-1, TIF file.

10.1523/ENEURO.0115-23.2023.f3-2 Extended Data Figure 3-2 Proportion of excluded videos from verification ranks 4 and 5 as described in Figure 3*C*,*D*. The proportion of videos excluded did not differ significantly when grouped by treatment (***A***) or stimuli type (***B***). Download Figure 3-2, TIF file.

### Statistical analysis

For all analyses, the entire 5 min of the sample or test phase was analyzed. Total stimuli exploration times were calculated by taking the sum of the time spent interacting with each stimulus, as measured in seconds. A discrimination ratio (DR) was calculated for each test session, which reflects the time spent with the novel stimulus compared with the average time spent with the familiar stimuli. This metric is calculated by the equation DR = [T (novel) – T (avg. familiars)/T (total)], and produces a ratio between −1 and +1, that indicates a familiar and novelty preference, respectively. A DR was also calculated for interaction bout count, while untransformed values were used to assess distance traveled and novel approach latency. Rats were excluded from the final analysis if all stimuli in the box were not visited in the sample phase, if an item was knocked over or moved, or if the video was blurry. From the test establishment experiments, two male rats were removed from the 3-object IST, 1 from the 3-odor IST, 1 from the 3-odor DST, and 1 from the 6-odor IST. Because of missing video footage, 8 values are missing from each 3-object and 6-object IST and DST sample phase mean ± SEM calculations. From the acute *Cannabis* exposure interaction bout duration data, six videos were excluded from the 6-object IST, two from the 6-object DST, one from the 6-odor IST, and two from 6-odor DST. From the bout count data, sevevn were excluded from the 6-object IST, three from the 6-object DST, and two from 6-odor DST.

Data were analyzed using GraphPad Prism 8.0.1 software. To evaluate the DRs generated from interaction times in the test validation and establishment experiment, one-sample *t* tests were used against chance (i.e., 0). To evaluate the total exploration times in the test validation and establishment experiment, two-way ANOVAs (followed by Bonferroni’s multiple comparisons test) with factors of Phase (sample vs test) and Item Count (3 vs 6) were used. To evaluate the total exploration times following *Cannabis* smoke exposure, two-way ANOVAs (followed by Bonferroni’s multiple comparisons test) with factors of Phase (sample vs test) and Treatment [Air Control vs high-THC (Skywalker) vs high-CBD (Treasure Island)] were used. Following *Cannabis* exposure, to evaluate the DRs and untransformed values measuring interaction time, bout count, distance traveled, and novel approach latency, one-way ANOVAs (followed by Turkey’s multiple comparisons test) with a factor of Treatment (Air Control vs high-THC vs high-CBD) were used. Lastly, to evaluate the interaction time DRs (novelty preference) against chance, one-sample *t* tests against 0 were used; *p* values that were less than or equal to 0.05 were considered significant.

## Results

### Male rats perform both the IST and DST with objects and odors, using either 3-stimuli or 6-stimuli

The 3-object and 6-object IST and DST were validated for male rats by adopting protocols similar to those used with mice (Sannino et al., 2012; Olivito et al., 2016, 2019). Male rats spent significantly more time with the novel object in comparison to the familiar objects in the 3-object IST (*t*_(14)_ = −6.29, *p* < 0.001), and in the 6-object IST (*t*_(14)_ = −5.02, *p* < 0.001; Fig. 1*E*). Male rats also displayed novelty preference in the 3-object DST (*t*_(16)_ = −5.09, *p* < 0.001), and in the 6-object DST (*t*_(14)_ = −3.94, *p* < 0.001; Fig. 1*E*). A comparison of the IST and DST DRs showed no differences between the 3-object (*t*_(30)_ = 0.98, *p* = 0.36) or 6-object (*t*_(28)_ = 1.40, *p* = 0.17) variations (Fig. 1*E*). All treatment groups performed better than chance (*t*_(15)_ = 7.35, *p* < 0.0001 (3-object IST); *t*_(14)_ = 8.41, *p* < 0.0001 (6-object IST); *t*_(15)_ = 8.52, *p* < 0.0001 (3-object DST); *t*_(14)_ = 7.31, *p* < 0.0001 (6-object DST; Fig. 1*E*).

A significant effect of Phase was seen on the total stimuli interaction time in the IST with objects (*F*_(1,39)_ = 9.63, *p* = 0.004), with no effect of Item Count (*F*_(1,39)_ = 1.62, *p* = 0.21) or an interaction (*F*_(1,39)_ = 0.11, *p* = 0.74) present (Table 1). Male rats spent more time exploring stimuli in the sample phase of the object IST than the test phase. There was also a significant effect of Phase on the total stimuli interaction time in the object DST (*F*_(1,39)_ = 13.89, *p* = 0.0006), with no effect of Item Count (*F*_(1,39)_ = 3.78, *p* = 0.059) or an interaction (*F*_(1,39)_ = 2.61, *p* = 0.11) present (Table 1). Inspection of the data revealed that in the object DST, male rats spent more time exploring stimuli in the sample phase than the test phase.

**Table 1:** Summary of all interaction times for validation of the tests summarized in **Figure 1**

	Object IST		Object DST		Odor IST		Odor DST	
	Sample*	Test*	Sample*	Test*	Sample	Test	Sample	Test
3 Item	71.45 ± 12.1	47.98 ± 6.5	68.43 ± 13.4	104.43 ± 18.9	31.99^#^ ±7.3	58.23 ± 5.3	35.69 ± 8.4	54.74 ± 5.9
6 Item	63.50 ± 5.4	34.30 ± 4.1	47.06 ± 5.	50.39 ± 6.9	38.14^#^ ±7.6	38.59 ± 5.2	33.83 ± 6.3	38.20 ± 3.6

The mean (±SEM) for the total interaction time seen with stimuli is recorded for each sample and test phase in the IST and DST with objects or odors. * Significant main effect of Phase on object IST and DST (*p* < 0.05). # Significant effect of Item Count on exploration times in the sample phase of the odor IST (*p* = 0.047).

In the tests with odors, male rats also showed novelty preferences in the 3-odor and 6-odor IST and DST (Fig. 1*F*). Male rats spent significantly more time with the novel odor compared with the familiar odors in the 3-odor IST (*t*_(7)_ = −1.87, *p* < 0.05) and 6-odor IST (*t*_(10)_ = −6.59, *p* < 0.001; Fig. 1*F*). Novelty preference was also demonstrated in the 3-odor DST (*t*_(6)_ = −7.94, *p* < 0.001), and in the 6-odor DST (*t*_(11)_ = −3.92, *p* < 0.01; Fig. 1*F*). Lastly, no differences between the IST and DST DRs were found in the 3-odor (*t*_(13)_ = −1.44, *p* = 0.17) or 6-odor (*t*_(21)_ = 1.60, *p* = 0.12) variations (Fig. 1*F*). All treatment groups performed better than chance [*t*_(7)_ = 5.04, *p* = 0.0015 (3-odor IST); *t*_(11)_ = 7.36, *p* < 0.0001 (6-odor IST); *t*_(7)_ = 5.40, *p* = 0.0010 (3-odor DST); *t*_(11)_ = 10.61, *p* < 0.0001 (6-odor DST); Fig. 1*F*].

In the odor IST, there was no effect of Phase on the total stimuli interaction time (*F*_(1,36)_ = 1.16, *p* = 0.29), but a main effect of Item Count (*F*_(1,36)_ = 4.55, *p* = 0.040) and a significant interaction was present (*F*_(1,36)_ = 4.24, *p* = 0.047; Table 1). Male rats spent more time exploring odors in the sample phase of the 6-odor IST than in the 3-odor IST (*p* = 0.031). In the odor DST, there was no main effect of Phase (*F*_(1,36)_ = 2.34, *p* = 0.14), Item Count (*F*_(1,36)_ = 3.79, *p* = 0.06) or an interaction (*F*_(1,36)_ = 1.49, *p* = 0.23) present (Table 1).

### Combining automated and human stopwatch scoring is a valid behavioral quantification approach

To quantify rat behavior following *Cannabis* smoke exposure using the hybrid scoring method, we created a video set of 288 test phase videos of the 6-stimuli test variations. Sample phase videos were all manually scored, where inclusion criterion was applied as described above, and included test phase videos were analyzed using our automated behavioral quantification pipeline.

To assess the accuracy of model predictions for both pose-estimation and behavioral classification, we used software native performance metrics that compare machine-generated predictions to manual annotation. The spatial coordinates of human annotated and machine-predicted points-of-interest differed by a mean Euclidian distance of 4.89 pixels on videos within the model training set and 4.35 pixels on test videos. Pose-estimation quality was further assessed by calculating the average prediction confidence for each point-of-interest by video (Extended Data Fig. 2-1). We found that the average prediction confidence ranged between 92.8% and 97.4% by point-of-interest, where no significant differences were observed between object-based and odor-based videos. Behavioral classifier performance was evaluated by a series of confusion matrices (Fig. 2*D*,*E*) that report the precision, recall, and combined F1 score for each model. In short, both classifiers demonstrate high precision and recall (object F1 = 0.927, odor F1 = 0.897) when assessed by comparing manual annotation to classifier predictions on randomly selected test video frames. However, when classifier performance was assessed by comparing predictions on randomly selected interaction bouts, object classifier performance changed marginally (F1 = 0.93), but odor classifier performance decreased markedly (F1 = 0.63). For both the object and odor classifiers, the behavioral features most heavily weighted for model predictions include distance to stimuli, nose movements, region-of-interest, and spatial dynamics between points-of-interest (Fig. 2*C*). Additional details regarding model training and assessments can be found in the Extended Data.

To verify the reliability of supervised machine learning-generated predictions relative to traditional stopwatch-based and automated region of interest-based scoring, we conducted a three-way correlational analysis on generated interaction DRs (Fig. 3*A*,*B*). We found that, across stimuli, supervised machine learning-generated predictions were more highly correlated with human stopwatch scoring than region of interest-based scoring; however, supervised machine learning-generated predictions were more highly correlated with human stopwatch scoring for object interaction (*r* = 0.75) relative to odor interaction (*r* = 0.53). Additionally, we found that, across stimuli, region of interest-based scoring held a weaker correlation relative to both human stopwatch scoring (object: *r* = 0.42, odor: *r* = 0.28) and supervised machine learning-generated (object: *r* = 0.45, odor: *r* = 0.42) interaction DRs. To account for instances where supervised machine learning predictions significantly differ from human stopwatch scoring, we created a five-tiered verification rank system, where supervised machine learning-generated predictions on videos with ranks four or five were replaced by human stopwatch scoring for the final analysis (Fig. 3*C*). Upon visual inspection of supervised machine learning-generated predictions, we found that ∼80% of object-based videos met inclusion criteria, while only ∼60% of odor-based videos met inclusion criteria (Fig. 3*D*). To justify supplementing human stopwatch scoring for suboptimal supervised machine learning-generated predictions, we conducted a correlational analysis between human stopwatch scoring and supervised machine learning interaction DRs only on videos which met inclusion criteria. We found that human stopwatch scoring and supervised machine learning interaction DRs were moderately-to-highly correlated (Fig. 3*E*, *r* = 0.83, Fig. 3*F*, *r* = 0.87) across stimuli type.

### High-THC, but not high-CBD, *Cannabis* smoke exposure impairs novelty preference for high-memory (DST) loads with object stimuli

Interaction bout duration DRs were investigated to examine whether novelty preference was impacted by treatment within each test variation. No effect of Treatment in the 6-object IST (*F*_(2,61)_ = 0.85, *p* = 0.43) was found (Fig. 4*C*). Using an analysis of the raw effect sizes, there were no notable effect sizes to report (Table 3). A main effect of Treatment was present in the 6-object DST (*F*_(2,63)_ = 3.75, *p* = 0.03), with a significant difference seen between the Air Control and high-THC groups after a Tukey’s multiple comparisons test (*p* = 0.04; Fig. 4*C*). The difference between the Air Control and high-THC groups represents a moderate effect size [*d* = −0.66, 95% confidence interval (CI) (1.27, −0.035), *p* = 0.03; Table 3]. Most treatment groups performed significantly better than chance (IST-Air Control: *t*_(23)_ = 3.15, *p* = 0.004; IST-high-THC: *t*_(19)_ = 2.24, *p* = 0.037; IST-high-CBD: *t*_(19)_ = 4.27, *p* = 0.0004; DST-Air Control: *t*_(18)_ = 3.29, *p* = 0.004; DST-high-CBD: *t*_(24)_ = 2.14, *p* = 0.042) except for the high-THC group in the 6-object DST (*t*_(22)_ = 0.66, *p* = 0.51; Fig. 4*C*).

We then investigated novel approach latency values, defined as the interval between rats being placed into the experimental arena and interacting with the novel object. No effect of Treatment on novel approach latency values was observed in either the 6-object IST (*F*_(2,70)_ = 0.77, *p* = 0.46) or the 6-object DST (*F*_(2,67)_ = 0.076, *p* = 0.93; Fig. 4*D*). Next, to examine whether male rats visited the novel object at a higher frequency than familiar objects, we evaluated the interaction bout DRs (Fig. 4*E*). Here, we showed a significant main effect of Treatment in the 6-object IST (*F*_(2,64)_ = 8.05, *p* < 0.001), as the Air Control (*p* = 0.001) and high-THC (*p* = 0.01) groups were different from the high-CBD group. However, we failed to find a main effect of Treatment on bout count DRs in the 6-object DST (*F*_(2,64)_ = 0.96, *p*= 0.39; Fig. 4*E*). Lastly, the impact of *Cannabis* smoke exposure on locomotion during memory testing was evaluated. We found no main effects of Treatment on distance in either the 6-object IST (*F*_(2,70)_ = 0.58, *p* = 0.56), or in the 6-object DST (*F*_(2,67)_ = 0.30, *p* = 0.74; Fig. 4*F*).

When assessing total stimuli interaction time, a main effect of Treatment (*F*_(2,129)_ = 4.07, *p* = 0.019), and of Phase (*F*_(1,129)_ = 6.45, *p* = 0.012) was seen in the 6-object IST, with no significant interaction (*F*_(2,129)_ = 0.49, *p* = 0.62; Table 2). In the 6-object DST, there was a main effect of Phase on total stimuli interaction time (*F*_(1,135)_ = 7.87, *p* = 0.0058), with no main effect of Treatment (*F*_(2,135)_ = 1.81, *p* = 0.17) or an interaction (*F*_(2,135)_ = 0.75, *p* = 0.47; Table 2). Following each smoke treatment, the number of boli was counted in the smoke exposure cage (Fig. 6). A main effect of Treatment was observed (*F*_(2,141)_ = 172.90, *p* < 0.0001), with a significant increase in the number of boli recorded following either Skywalker (*p* < 0.0001) or Treasure Island (*p* < 0.0001) smoke exposure after a Tukey’s multiple comparisons test. However, there was no difference in the number of boli observed between Skywalker or Treasure Island (*p* = 0.40) smoke exposure groups.

**Table 2:** Summary of all interaction times for tests with *Cannabis* summarized in **Figures 2-5**

	Object IST		Object DST		Odor IST		Odor DST	
	Sample*	Test*	Sample^#^	Test^#^	Sample^&^	Test^&^	Sample^%^	Test^%^
Air Control	36.21 ± 2.9	42.93 ± 4.0	35.61 ± 3.2	39.23 ± 3.4	37.75 ± 2.8	47.78 ± 5.8	39.16 ± 3.1	50.12 ± 5.6
High-THC	36.01 ± 3.7	46.90 ± 4.1	39.65 ± 3.5	49.72 ± 4.6	34.27 ± 3.1	57.94 ± 4.8	35.29 ± 2.8	55.27 ± 6.5
High-CBD	30.09 ± 3.0	33.97 ± 2.7	33.9 ± 3.1	46.96 ± 4.2	31.54 ± 2	36.93 ± 5.5	40.54 ± 3.4	48.03 ± 6.1

The mean (±SEM) for the total interaction time seen with stimuli is recorded for the sample and test phases in the different 6-object and 6-odor IST and DST across the Air Control, high-THC, and high-CBD treatment groups. * Significant effect of Treatment (*p* = 0.019) and of Phase (*p* = 0.012) on object IST. # Significant effect of Phase (*p* = 0.0058) on object DST. & Significant effect of Treatment (*p* = 0.025) and Phase (*p* = 0.0004) on odor IST. % Significant effect of Phase (*p* = 0.0019) on odor DST.

**Figure 5.**
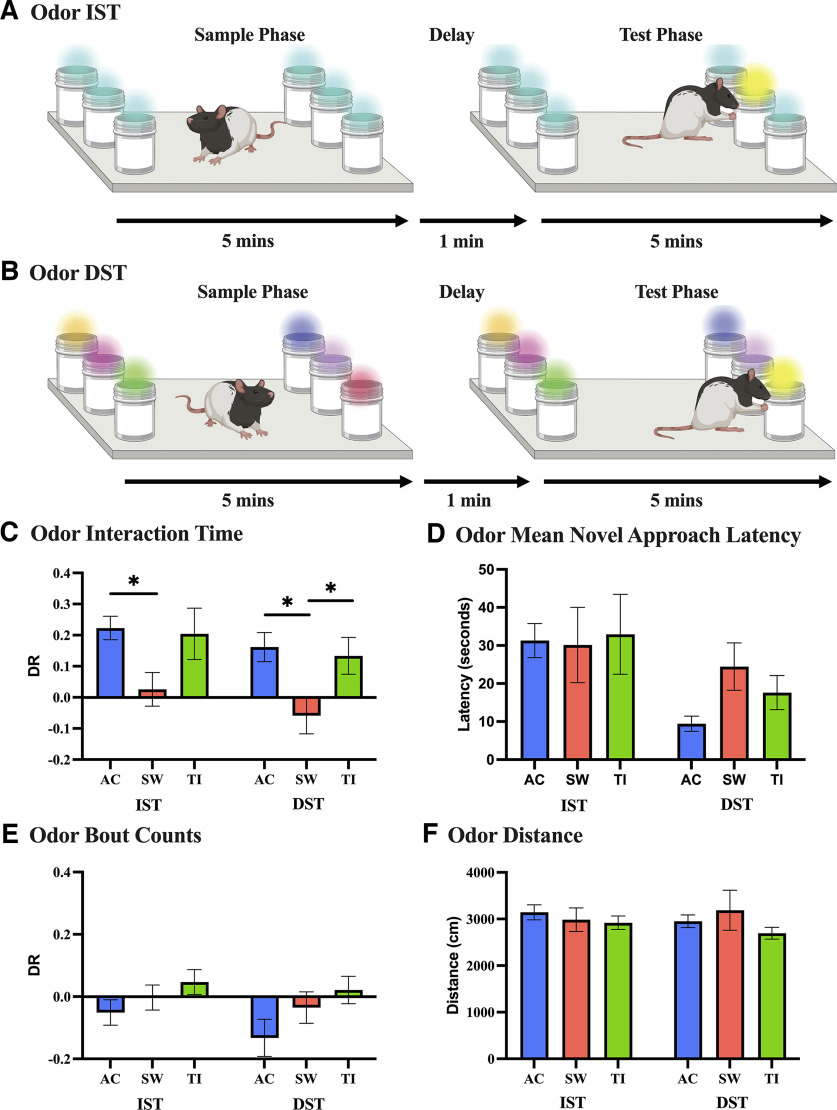
High-THC *Cannabis* smoke exposure impacts novelty preference under high-memory (DST) and low-memory (IST) loads using odor stimuli, with no impact on distance traveled, frequency of item visitation, or approach latencies. ***A***, Example IST with odors is visualized, showing six identical items in the sample phase, with a novel odor introduced after a 1-min delay in the test phase. ***B***, A DST with odors variation is shown, with an identical task progression, but instead starts with six different odors in the sample phase. **C** Interaction measured as time spent with an odor was generated using the human-machine hybrid scoring approach and visualized using a discrimination ratio for both variations using odor stimuli. In the 6-odor IST (*n* = 75), a significant decrease in novelty preference is seen in the AC group in comparison to the SW group (*p* = 0.046). Whereas in the 6-odor DST (*n* = 73), a significant decrease in novelty preference is seen in the SW group from both the AC (*p* = 0.023) and TI (*p* = 0.046) groups. ***D***, The mean novel approach latency in the 6-odor IST (*n* = 79) and 6-odor DST (*n* = 73) variations is shown to be consistent between treatment groups. ***E***, To illustrate the frequency of visitations to the novel odor in comparison to the familiar odors, bout counts are visualized using a discrimination ratio. No differences between treatment groups or 6-odor IST (*n* = 79) and 6-odor DST (*n* = 73) are seen. ***F***, Distance traveled (cm) in the 6-odor IST (*n* = 79) and 6-odor DST (*n* = 73) variations is comparable across treatment groups. Data represents mean ± SEM, *p < 0.05. Abbreviations: high-THC *Cannabis* smoke (SW); high-CBD *Cannabis* smoke (TI); Air Control (AC). This figure was created using BioRender.

**Figure 6.**
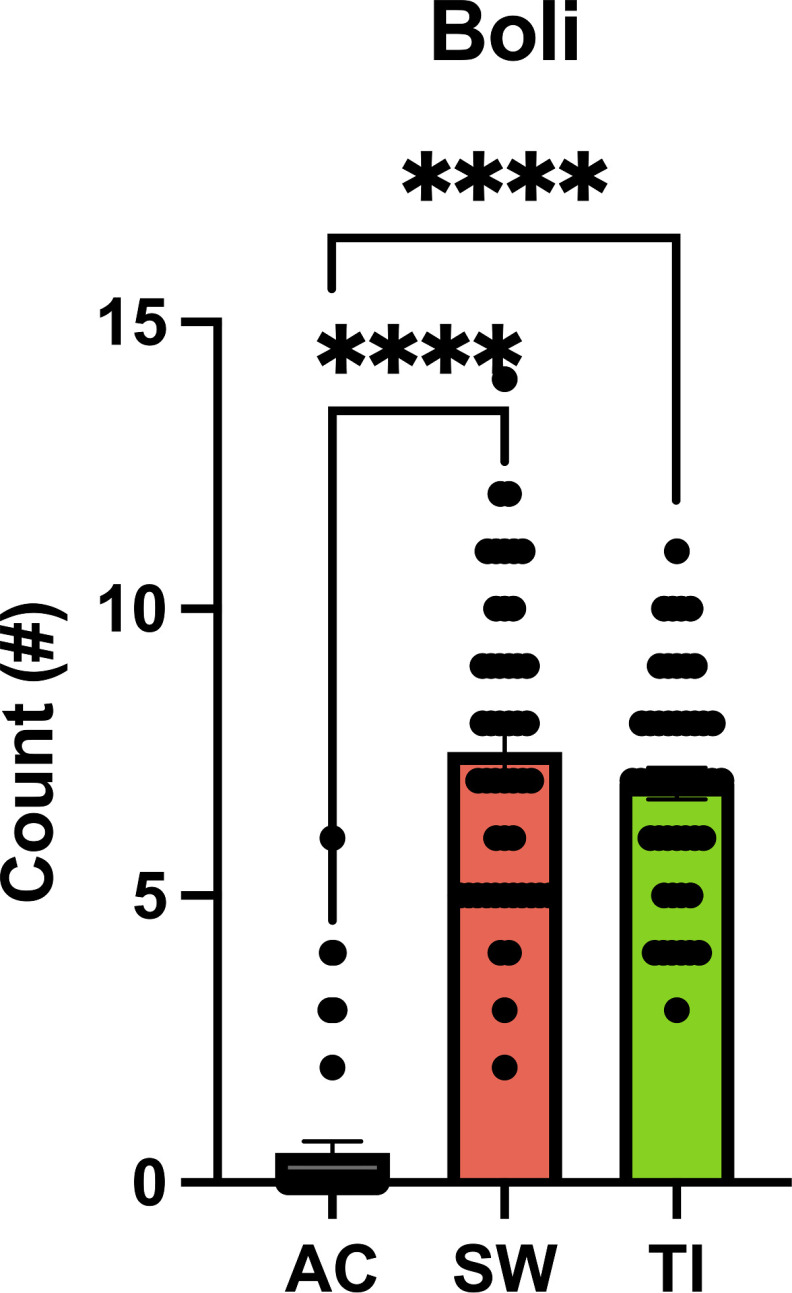
Boli count following smoke exposure treatment. A significant increase in the number of boli recorded was observed following *Cannabis* smoke exposure in comparison to the Air Control (AC) condition. However, no difference between Skywalker (SW) or Treasure Island (TI) groups was recorded. ****p < 0.001. high-THC Cannabis smoke (SW); high-CBD Cannabis smoke (TI); Air Control (AC).

### High-THC, but not high-CBD, *Cannabis* smoke exposure impairs novelty preference for high-memory (DST) and low-memory (IST) loads with odor stimuli

*Cannabis* smoke exposure impacted the interaction bout duration DRs in the IST and DST. An effect of Treatment in the 6-odor IST (*F*_(2,73)_ = 3.54, *p* = 0.034) was seen, with a significant difference present between the Air Control and high-THC groups (Tukey’s multiple comparisons test, *p* = 0.046; Fig. 5*C*). A moderate effect size was found between the high-THC and Air Control groups [*d* = −0.78, 95% CI (1.41, −0.19), *p* = 0.0058; Table 3]. A main effect of Treatment for interaction bout duration DRs was also present in the 6-odor DST (*F*_(2,71)_ = 4.3, *p* = 0.017), with a significant difference between the Air Control and high-THC groups (*p* = 0.024) and between high-THC and high-CBD groups (*p* = 0.046) after a Tukey’s multiple comparisons test (Fig. 5*C*). A moderate effect size was also found between the high-THC and Air Control groups [*d* = −0.87, 95% CI (1.47, −0.23), *p* = 0.0042; Table 3]. Air Control and high-CBD treatment groups performed significantly better than chance in both tests (IST-Air Control: *t*_(25)_ = 5.90, *p* < 0.001; IST-high-CBD: *t*_(22)_ = 2.47, *p* = 0.022; DST-Air Control: *t*_(23)_ = 3.45, *p* = 0.002; DST-high-CBD: *t*_(27)_ = 2.25, *p* = 0.033), whereas the high-THC group did not in either the 6-odor IST (*t*_(26)_ = 0.47, *p* = 0.64) or 6-odor DST tests (*t*_(21)_ = 1.00, *p* = 0.33; Fig. 5*C*). There was no effect of Treatment in the 6-odor IST (*F*_(2,77)_ = 0.036, *p* = 0.70), or in the 6-odor DST (*F*_(2,71)_ = 0.87, *p* = 0.42) when investigating novel approach latency (Fig. 5*D*). Interaction bout DRs were also determined to be unaffected by *Cannabis* exposure with no effect of Treatment in the 6-odor IST (*F*_(2,77)_ = 1.46, *p* = 0.24), and the 6-odor DST (*F*_(2,70)_ = 2. 19, *p*= 0.12; Fig. 5*E*). Treatment also did not impact the distance traveled by male rats in either the 6-odor IST (*F*_(2,77)_ = 0.36, *p* = 0.70), or in the 6-odor DST (*F*_(2,71)_ = 0.87, *p* = 0.42; Fig. 5*F*).

**Table 3:** Summary of the effect sizes (Cohen’s *d*) and corresponding *p*-values for Figures 4*C* and 5*C*

	AC-SW Cohen’s *d*	AC-SW *p* value	AC-TI Cohen’s *d*	AC-TI *p* value
6-Object IST	−0.25 [95.0%CI −0.856, 0.357]	0.409	0.291 [95.0%CI −0.323, 0.872]	0.319
6-Object DST	−0.655 [95.0%CI −1.27, −0.035]	0.03*	0.118 [95.0%CI −0.507, 0.716]	0.7
6-Odor IST	−0.783 [95.0%CI −1.41, −0.194]	0.0058**	0.0239 [95.0%CI −0.539, 0.637]	0.936
6-Odor DST	−0.874 [95.0%CI −1.47, −0.228]	0.0042**	−0.172 [95.0%CI −0.727, 0.413]	0.544

The unpaired Cohen’s *d* [confidence interval lower bound, upper bound] for interaction times seen between novel and familiar stimuli is recorded for the test phases in the 6-object and 6-odor IST and DST across the Air Control, high-THC, and high-CBD treatment groups. *p < 0.05, **p < 0.01, ***p < 0.001.

For exploration times in the 6-odor IST, a main effect of Treatment (*F*_(2,142)_ = 3.78, *p* = 0.025), and of Phase (*F*_(1,142)_ = 12.90, *p* = 0.0004) was seen, with no significant interaction (*F*_(2,142)_ = 2.27, *p* = 0.11; Table 2). Male rats spent more time exploring stimuli in the Air Control sample phase than in the high-THC test phase (*p* = 0.017). As well, male rats explored stimuli more in the sample phase than in the test phase following high-THC (*p* = 0.0035), while spending more time exploring stimuli in the test phase following high-THC smoke exposure than following high-CBD smoke exposure (*p* = 0.009). In the 6-odor DST, there was a main effect of Phase on total stimuli interaction time (*F*_(1,134)_ = 10.01, *p* = 0.0019), with no main effect of Treatment (*F*_(2,134)_ = 0.021, *p* = 0.98) or an interaction (*F*_(2,134)_ = 0.85, *p* = 0.43). Inspection of the data revealed that male rats spent more time exploring the odors during the test phase of the 6-odor DST, regardless of Treatment (Table 2).

## Discussion

In the present study, we showed that male rats display novelty preferences in both the IST and DST with three and six objects, similar to previous findings using objects in male mice (Sannino et al., 2012; Olivito et al., 2016, 2019). We also demonstrate, for the first time, that male rats exhibit novelty preference with three and six odor stimuli, as measured in the IST and DST (Fig. 1). Overall, male rats spent more time exploring stimuli in the sample phases of the 6-item IST and DST compared with the test phases, with stimuli-specific differences (Table 1). Following high-THC *Cannabis* smoke exposure in the tests with objects, a significant decrease in novelty preference was seen in the 6-object DST, but not in the 6-object IST (Fig. 4*C*). However, for odor-based tests, we observed novelty preference impairments for high-memory and low-memory loads (Fig. 5*C*). No notable treatment effect on total stimuli exploration time was present in the 6-object IST, but a significant increase in stimuli exploration time was seen in the test phase of the 6-object DST for all treatments (Table 2). In the 6-odor IST, male rats explored stimuli less in the sample phase compared with the test phase following high-THC *Cannabis* smoke exposure, with no notable effects in the 6-odor DST (Table 2). Taken together, these findings suggest that *Cannabis* smoke exposure impacts novelty preference in male rats in a load-dependent and stimuli-specific manner.

### Male rats demonstrate novelty preference in both the IST and DST with objects and odors

In the test validation experiment, male rats demonstrated pronounced novelty preference in all test variations (Fig. 1). The preferential interaction with novel stimuli compared with familiar stimuli after a brief delay suggests that recognition memory is intact in both object and odor-based tests (Shrager et al., 2008; van Vugt et al., 2010; Sannino et al., 2012). The varying memory loads between the IST and DST also present the opportunity to examine incidental memory capacity (Shrager et al., 2008; Sannino et al., 2012). In this study, 3-item and 6-item tests were run to replicate Sannino et al.’s (2012) results showing that male mice demonstrated novel object discrimination when using up to 6 objects. To enable direct comparisons between object and odor stimuli, sets of three odors and 6 odors were chosen as well. Male rats explored the object stimuli a comparable amount between test variations and with varying numbers of stimuli (Table 1). Male rats did, however, spend significantly less time exploring objects in the test phase of the 6-object DST compared with the sample phase (Table 1). As the test phase progressed, male rats would have had increasing familiarization with all items in the test phase, which may explain the decreased total exploration times (Broadbent et al., 2010). Interestingly, there were no notable differences in the total stimuli interaction times between the 3-odor and 6-odor variations, indicating that while the total time male rats spent exploring stimuli was the same, the time spent exploring each individual stimulus in the 6-item variation was about half of that for the 3-item variation (Table 1). In future experiments, it would be interesting to assess novelty preferences and exploration preferences in test with more than six stimuli, as has been reported for objects in male mice (Sannino et al., 2012). As well, these tests must be validated for use in female rats. Recent findings show sex differences in delay-dependent incidental memory capacity for objects in mice, which may depend on subcortical inhibitory control of the hippocampus (Torromino et al., 2022). These findings in mice raise the possibility that similar sex differences exist in rats, a question that will be investigated in future experiments. Validating the odor-based spontaneous tests in male and female mice would also be worthwhile given their affordability and availability of genetic models.

The IST and DST allow the study of novelty preferences for stimuli arrays of varying size in a spontaneous, simple, and cost-effective manner. The tests do not require rodents to apply learned rules or procedures, eliminating the need for extensive training or researcher involvement. The tests also evoke minimal stress in rodents and do not require typical food-restriction protocols to increase reward-driven performance. Performance on the object tests likely engage a combination of visual and tactile recognition memory, but as the object stimuli were constructed with LEGO blocks of similar size, identical smooth textures, and sharp corners, the tests were likely biased to engage visual recognition memory. The object-based test may engage visual, perirhinal, medial prefrontal, parietal, and entorhinal cortices, as well as the hippocampus and thalamus to enable the object-based recognition memory across a delay (Hannesson et al., 2004; B.D. Winters et al., 2004; Fernández and Tendolkar, 2006; Barker et al., 2007; Dere et al., 2007; Cazakoff and Howland, 2011; Churchwell and Kesner, 2011; Peters et al., 2013; Sugita et al., 2015; Creighton et al., 2018). The odor stimuli primarily engage odor-based recognition as identical opaque glass jars were used in the tests. A circuit including piriform, entorhinal, medial prefrontal, and orbitofrontal cortices, along with hippocampus may be involved in the odor-based memory across a delay (Ramus and Eichenbaum, 2000; Alvarez and Eichenbaum, 2002; Mouly and Sullivan, 2010; Davies et al., 2013; Sandini et al., 2020). To examine the brain regions and neural mechanisms underlying working memory capacity in different contexts, a variety of behavioral tasks have been employed. Visuospatial working memory and working memory capacity are examined with the radial-arm maze, Barnes Maze, and operant delayed nonmatching-to-sample and delayed-match-to-sample tasks (Kirchner, 1958; Daneman and Carpenter, 1980; Dudchenko, 2004; Cowan, 2010; Dudchenko et al., 2013; Oomen et al., 2013; Wilhelm et al., 2013; Vorhees and Williams, 2014; Scott et al., 2020; Barnard et al., 2022). To study odor based working memory capacity, the odor span task and other tests that employ a nonmatch-to-sample-rules have often successfully been used (Dudchenko et al., 2000; Scott et al., 2020). Although these tasks measure working memory capacity, they require food restriction, extensive training, and heavy researcher involvement. Spontaneous recognition tests circumvent these weaknesses, although the cognitive processes involved in incidental memory capacity may differ from those necessary for more goal-directed forms of working memory capacity.

### High-THC, but not high-CBD, *Cannabis* smoke exposure impairs novelty preferences for both object and odor stimuli

To evaluate the effects of *Cannabis* smoke exposure on incidental memory over short delays, we used the hybrid scoring approach to assess novelty preference in the IST and DST with objects and odors. The 6-item object and odor tests were selected as they would be expected to engage circuits related to capacity, while still ensuring reliable performance in control groups, as previously established in mice (Sannino et al., 2012; Torromino et al., 2022). Novelty preference was primarily inferred from interaction bout duration, as it was not predicted by interaction bout count or novel approach latency. Following high-THC *Cannabis* smoke exposure in the tests with objects, a significant decrease in novelty preference was seen in the 6-object DST, but not in the 6-object IST (Fig. 4*C*). For odor-based tests, an impairment in novelty preference was observed in both the IST and DST following high-THC *Cannabis* smoke exposure (Fig. 5*C*). In all tests, novelty preference was similar between the Air Control and high-CBD *Cannabis* smoke groups. Additionally, no differences in locomotion were observed among treatment groups. The increased total stimuli exploration time in the sample phases of the object DST compared with the test phases likely indicates familiarity with the items in the test phase that were previously presented during the sample phase (Broadbent et al., 2010). Interestingly, in the 6-odor IST, there was lower stimuli exploration time in the sample phase compared with the test phase following high-THC *Cannabis* smoke exposure (Table 2).

Overall, the deficits in novelty preference following high-THC *Cannabis* smoke exposure in both the object and odor-based tests in male rats are likely attributable to the actions of THC, and not to smoke alone. Interestingly, boli excretion was increased following acute *Cannabis* smoke exposure, but with no differences observed between the high-THC and high-CBD groups (Fig. 6). As novelty preference was comparable between the Air Control and high-CBD groups, smoke likely did not provoke stress-induced performance deficits. As behavioral testing was conducted 20 min following the initiation of *Cannabis* smoke exposure, plasma and brain THC concentrations would have been near their peak in the rats (Hložek et al., 2017; Ravula et al., 2019; Baglot et al., 2021; Barnard et al., 2022; Moore et al., 2022). Analysis of plasma from male rats following an identical *Cannabis* smoke exposure paradigm revealed levels of 14.55 ± 1.59 ng/ml with a small amount of CBD (1.98 ± 0.38 ng/ml) 30 min after smoke exposure (Barnard et al., 2022). After high-CBD smoke exposure, negligible amounts of THC were found in plasma, along with 4.47 ± 1.15 ng/ml of CBD (Barnard et al., 2022). Thus, the current smoke exposure protocol increases blood plasma levels of THC to the low end of what is typically observed in humans following *Cannabis* cigarette consumption (Huestis et al., 1992; Grotenhermen, 2003; Huestis, 2007; Ramaekers et al., 2009; Newmeyer et al., 2016; Moore et al., 2022). Although the THC plasma levels in male rats were comparably low, we still observed the impact of *Cannabis* exposure on memory. The different THC-induced novelty preference impairments seen in the male rats between objects and odors may be because of the varying neural circuits underlying stimulus perception and integration (Fernández and Tendolkar, 2006; Mouly and Sullivan, 2010; Eriksson et al., 2015; Constantinidis and Klingberg, 2016; Galizio, 2016). Under low memory loads (IST), treatment does not impact object novelty preference, consistent with unperturbed working memory performance previously observed in a 2-item novel object recognition (NOR) test following chronic exposure to 5.6% THC *Cannabis* cigarettes (Bruijnzeel et al., 2016). The novelty preference deficits observed following high-THC *Cannabis* exposure in the 6-odor IST also might have been affected by the decreased exploration time in the sample phase. Lastly, the similar THC-induced deficits in the DST with objects and odors could be because of sensitivity of the working memory subconstructs evoked under high memory loads to *Cannabis* exposure (Barch and Smith, 2008).

### The case for, and caveats of, supervised machine learning-based behavioral analysis at scale

Automated behavioral analysis represents a potential paradigm shift in the way behavioral data are generated and shared (Mathis et al., 2020). In the present study, we demonstrate the case for, and caveats of, using a supervised machine learning-based analysis method for complex behavior at scale. In short, pose-estimation data were used to train two behavioral classifiers to predict interaction events with objects and odors. To assess the reliability of supervised machine learning-generated behavioral predictions, we compared quantified rat-stimulus interaction to human stopwatch and region of interest-based scoring. We found that supervised machine learning-generated predictions were more strongly correlated with human stopwatch than region of interest-based scoring; however, we observed that supervised machine learning-generated predictions were more highly correlated with human stopwatch-based scoring for object stimuli than for odor stimuli. As a methodological validation control, we conducted an inter-rater variability analysis to ensure that comparison of human stopwatch and supervised machine learning behavioral scoring is generalizable to manual scorers of varying experience levels (Extended Data Fig. 3-1). In short, we found a strong correlation between scorers of all experience levels (0.85 < *r* < 0.94), but a comparatively weaker correlation between experienced and beginner scorers. While a generally strong correlation between all scorers reinforces human stopwatch scoring as a gold-standard, experience-dependent changes in scoring accuracy underscore the value of high-throughput and objective scoring methods, such as the supervised machine learning-based method employed in this study.

Upon visual inspection of supervised machine learning-generated predictions, a near 30% increase in the proportion of excluded supervised machine learning-based odor interaction DRs is striking given that each classifier was trained on the same number of training frames, used identical algorithmic hyperparameters, and no significant treatment differences were observed in the proportion of excluded videos (Extended Data Fig. 3-2). We propose that this difference may be explained by divergent operational definitions of interaction in object and odor tests. Rat-object events encompassed interaction along the entire height of the object, while rat-odor interaction was only counted at a narrow space around the lid of the mason jar. As we employed a two-dimensional (2D) pose-estimation approach, movements along the height of stimuli were not well captured, potentially leading to suboptimal predictions and grounds for exclusion. While classifiers trained on 2D pose-estimation data show reliability on classifying behaviors restricted to single-plane spatiotemporal movements, recent studies of complex behaviors, such as self-grooming, generally train classifiers on 3D pose-estimation data to better capture the entirety of a movement and to minimize occlusion (Marshall et al., 2021, 2022; Minkowicz et al., 2023; Newton et al., 2023). Said differently, our assumption is not that the manual scorer and algorithm are using fundamentally different patterns of rat movement to infer behavior, but rather that the human is able to innately infer 3D from a 2D video, which is an important clue for interaction with stimuli that is not well captured in the automated analysis. Finally, software native performance metrics for both behavioral classifiers closely mirror those reported in published studies using supervised machine learning-based analysis; however, manual verification of predictions revealed significant instances of misclassification (C. Winters et al., 2022; Newton et al., 2023). We contend that supplementing classifier performance metrics with correlational analysis and verification steps are best practices when conducting scaled automated behavioral analysis.

While a full review of best practices in automated behavioral analysis approaches is beyond the scope of this study and has been reviewed in detail by others (Mathis et al., 2018, 2020; Luxem et al., 2023), hardware and software optimization is critical for promoting model generalizability. First, to fully capture behaviors of interest, researchers using automated behavioral analysis should be cognisant of the angle, and number, of camera perspectives used during filming (Luxem et al., 2023). Additionally, it is essential to include a diversity of training examples during model training, as a high degree of diversity in a training set will lead to a high degree of generalizability for both pose-estimation (DeepLabCut) and subsequent supervised machine learning-based analysis (SimBA). For example, within the present study, differences in color contrast, filming angle, and resolution likely contributed to a lack of DeepLabCut model generalizability between videos filmed for test validation (Fig. 1) and *Cannabis* manipulation (Figs. 4, 5). Taken together, supervised machine learning-based analysis is a promising tool for behavioral neuroscience, but this approach still faces some significant limitations, and researchers should adhere to available best practices to maximize the reliability of behavioral measurements.

In conclusion, using novel spontaneous tests and a hybrid scoring method, the impact of acute exposure to high-THC or high-CBD *Cannabis* smoke on incidental memory was evaluated in male rats. We show impaired object-based novelty preference after high-THC, but not high-CBD, *Cannabis* smoke exposure under a high-memory load. As well, we show deficits in odor-based novelty preference following high-THC *Cannabis* smoke exposure under both low-memory and high-memory loads. Ultimately, these data indicate that *Cannabis* smoke exposure impacts novelty preference in a load-dependent, and stimuli-specific manner in male rats.

## Synthesis

Reviewing Editor: Mark Laubach, American University Decisions are customarily a result of the Reviewing Editor and the peer reviewers coming together and discussing their recommendations until a consensus is reached. When revisions are invited, a fact-based synthesis statement explaining their decision and outlining what is needed to prepare a revision will be listed below. The following reviewer(s) agreed to reveal their identity: Ryan McLaughlin.

Thank you for sending your work to eNeuro. Your manuscript received generally positive reviews. Both reviewers have asked for revisions to the manuscript. The second reviewer feels strongly that you need to include female animals in the study, and has asked for additional data in the revised manuscript. Please respond to this issue in your revised manuscript. If you are unable to collect new data to address this concern, then the manuscript will require revisions to state why only males were used in the study (in the Intro section of the manuscript) and to include “male rats” in the title, abstract, significance statement, and wherever you make claims about effects of THC on behavior.

Reviewer 2 wrote the following about this issue in the consultation session about your manuscript:

“My major issues my review indicated, is that they did not test any female subjects, which I feel should be mandatory given that sex should be considered as a biological variable for all studies these days, particularly ones involving model development or refinement. With that said, I am not insisting they do a full sex comparison or be fully powered to test for sex differences (however, observing sex differences in their endpoints of interest would only serve to further strengthen the manuscript and increase the significance of their findings).”

An additional issue from Reviewer 1 was summarized in the consultation session as follows:

“I would like to see more explicit descriptions of their rationale (why they did things the way they did) and interpretations of results (why it is currently imperfect, and how it can improved). This type of machine learning behavioral analysis is an important but rapidly evolving field, and these types of discussion will help people use this paper to make continued improvements in the technology.”

Please include a summary of your changes to address these comments about the rationale and interpretational limits of your study, especially your use of machine learning methods.

Here are the specific comments from the two reviewers:

Reviewer 1

In this manuscript, the authors present the results of a series of experiments to validate the use of DeepLabCut to develop a semi-automated machine learning approach for performing novel object recognition test in rats, which was implemented to show that acute inhalation exposure to High-THC cannabis (but not low-THC cannabis) produces deficits in discrimination ratio in this test.

In general, the experiments appear to be well-designed and the results are appropriately interpreted in the context of prior research and limitations. The findings have important potential implications for evaluation of novel object recognition in rodents, as well as learning and memory changes following acute cannabis exposure. However, issues with the manuscript and figures outlined below should be addressed to be suitable for publication.

(1) The manuscript has a lot of abbreviations, and would be more accessible if this number could be reduced. The figures have abbreviations that are not explained in the figure or legend, and could be substantially improved for readability by reducing abbreviation use and/or defining them in the Figure/legend. For example, in Figure 3 ROI and HS is abbreviated in the figure but not defined in the legend, whereas the abbreviation for ML and DR are used in both figure and legend without definition.

(2) The overall approach compares the supervised machine learning based analysis to human stopwatch, with the human results serving as the gold standard. However, they did not compare data between multiple human investigators, which themselves may be prone to error. It is possible that the supervised machine learning is as good as the average human user, but not a specific human user. The authors may consider having a subset of videos analyzed by a second human user as a methodological validation control.

(3) The authors appropriately noted that the percentage of human-supplemented data was different between the two type of tests (odor and object). However, it was unclear if supplementation was equally distributed between the 3 biological groups (Air, Low-THC, High-THC) or if the experimental outcome may be biased due to changes in machine learning/human data ratio.

(4) There is no explanation for why the 3-object test was compared with the 6-object test. Additionally, no rationale was given for why the 6-object test was selected for subsequent studies based on these results.

(5) “Within the present study, our DLC model did not generalize well to behavioral videos filmed for task validation, likely due to divergent resolution dimensions and color contrast that was not well represented in the model training set.” Given that 20-40% of videos did not meet criteria, a more thorough discussion of how this could be improved is warranted. Is this a limitation of equipment/software/computing power? Could this have been improved by using different user-defined analysis parameters? This would help readers understand which technological advances could improve these approaches in the future as they implement this approach to their experiments.

(6) Figure 3D may be more useful as % (rather than #).

(7) Figure 4F and 5F should read “Object Interaction Time” for clarity

(8) The text in figure 3 is small and difficult to read

Reviewer 2This study aimed to investigate the impact of acute exposure to high-THC or high-CBD Ccnnabis smoke on working memory capacity (WMC) in rats. WMC was inferred by novelty preference after a short delay in spontaneous recognition-based tests using object or odor-based stimuli in different variations of the tests that present identical (IOT) and different (DOT) sets of stimuli for low- and high-cognitive loads, respectively. These tasks were developed and validated in the current study and were subsequently used to investigate the primary research question. A human-machine hybrid behavioral quantification approach was used, supplementing supervised machine learning-based classification with stopwatch-based manual scoring methods. The results indicate that high-THC, but not high-CBD, cannabis smoke exposure impaired novelty preference for objects under a high-cognitive load. Odor-based recognition deficits were seen under both low- and high-cognitive loads only following high-THC smoke exposure. Overall, the study provides evidence that THC-dominant cannabis smoke exposure impacts novelty preference, with potential implications for working memory deficits.

The manuscript has several strengths. It is very well written, clear and logical, the statistical analyses are appropriate and well described, and the interpretation of the data are reasonable. The development of a new task for measuring novel object preference/working memory deficits in rats is of broad interest to the field. The use of two different cannabis preparations (high THC vs. high CBD) is a major strength and the cannabis delivery system provides perhaps a more translational way to study impacts of cannabis compared to more traditional routes of administration. Additionally, the hybrid scoring approach on low confidence videos seems like an appropriate strategy since there can be issues with machine learning on edge cases or behaviors that are more ambiguous, as is likely the case with their odor-based task.

However, these strengths were outweighed by one major weakness - they did not test females and make no efforts to explain why females were not included in this study (??). Today, sound justification is required for not considering sex as a biological variable, and I feel this holds especially true for model development papers like this one. The authors should run female rats through this task before it be re-considered for publication. I strongly urge the researchers to consider conducting the work female rats and resubmitting, because the paper is otherwise strong, the findings are intriguing, and it has potential to offer a valuable contribution to the literature.

Additional minor/moderate concerns are provided below.

- The authors do not discuss the effect of high-CBD cannabis smoke in Fig. 4E. Is there a reason/explanation for why there was this particular difference between Treasure Island and Air Control?

- Is there really a “cognitive load” associated with this task? Is that really what this should be operationalized as? There is no consequence, positive or negative, to performing “well” on this task since there is no reinforcement for visiting the novel object/odor (i.e., it is ‘spontaneous’). Instead, the investigation of novel objects is the goal in this task, but that relies on the assumption that all objects and odors have equal valence, which I think is questionable. Could any of these odors be inherently aversive that would in turn lead to a bias in investigation? Did the investigators test inherent preference to odors or objects before beginning the study to ensure they were all generally equal? How do the investigators know that rats aren’t just going to a preferred object or odor consistently on a trial-by-trial basis? For example, if I completely lacked short-term/working memory, I would still avoid a smell of rotten eggs on every trial regardless of whether I am recalling information learned from my previous interaction or not. How can innate preference be dissociated from working memory performance? Additionally, why did the authors choose to manipulate “cognitive load” with the number and type of objects (3 vs. 6; IOD vs. DOT) as opposed to increasing the load on working memory by increasing the interval between trials? It seems like the impact of high-THC cannabis smoke exposure is on novel object preference specifically, which may or may not imply a working memory deficit. In this regard, I feel like the title is somewhat misleading.

- It would have been ideal to have measured circulating levels of THC, CBD, and metabolites in rats from this study in order to examine relationships between circulating cannabinoids and task performance. The citation provided for plasma levels in a similar study is helpful but indicates relatively low concentrations of THC and CBD. Do the authors have any speculations regarding contributions of other phytocannabinoids or terpenes to these effects, given that they are using whole-plan cannabis products?

- Were there any overt signs of distress or stress associated with forced smoke exposure? Did the authors measure fecal boli or any behavioral indices of stress in response to smoke exposure (avoidance, excessive grooming, etc.)? Was there any indication that one type of smoke was more stressful than the other?

- On page 16 of the discussion, the authors indicate that the lack of effect on NOR test performance following cannabis smoke exposure observed by Bruijnzeel et al. (2016) was potentially a power issue. However, I doubt this is the case because the same group has published two papers since this one, both using cannabis smoke and showing improvements in a delayed working memory task (Blaes et al., 2019; Hernandez et al., 2021).

- Overall I like hybrid scoring approach the authors adopted, but I would like to see a short discussion of what they think the human scorers are picking up that their classifiers are not. Is it the subtlety in movements of the head when rats are interacting that we can pick up on that the model struggles with since there are just 3 tracking points on the head? The authors state the criteria for training their classifiers, and presumably it’s similar to or the same as what human scorers are looking for, but a bit more discussion on the possible source of the discrepancy would be helpful. It could be helpful to see a few labeled odor videos with low correlation between human and machine-learning scoring with labels of when investigation is being measured by each.
