# Supplementary figures and images for: High-THC Cannabis Smoke Impairs Incidental Memory Capacity in Spontaneous Tests of Novelty Preference for Objects and Odors in Male Rats
Source: eNeuro. 2023 Dec 6;10(12):ENEURO.0115-23.2023. doi: 10.1523/ENEURO.0115-23.2023 (PMC10714893; doi:10.1523/ENEURO.0115-23.2023)

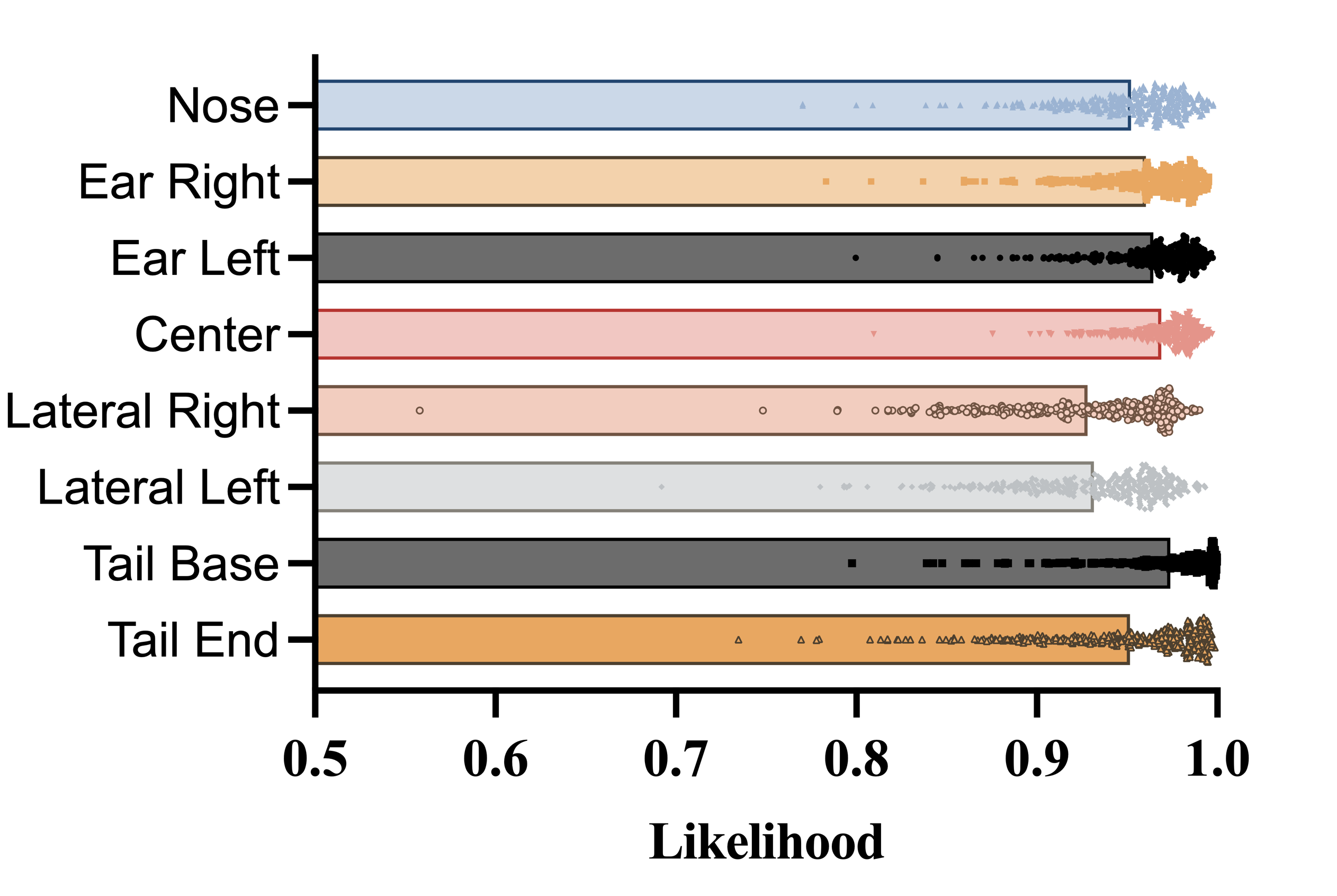

Supplement: Extended Data Figure 2-1 — Mean tracking confidence for each point-of-interest, by video. To calculate the mean tracking confidence for each video, the average of the likelihood column associated with each point of interest was calculated. Download Figure 2-1, TIF file. [file enu-eN-NWR-0115-23-s02.tif]

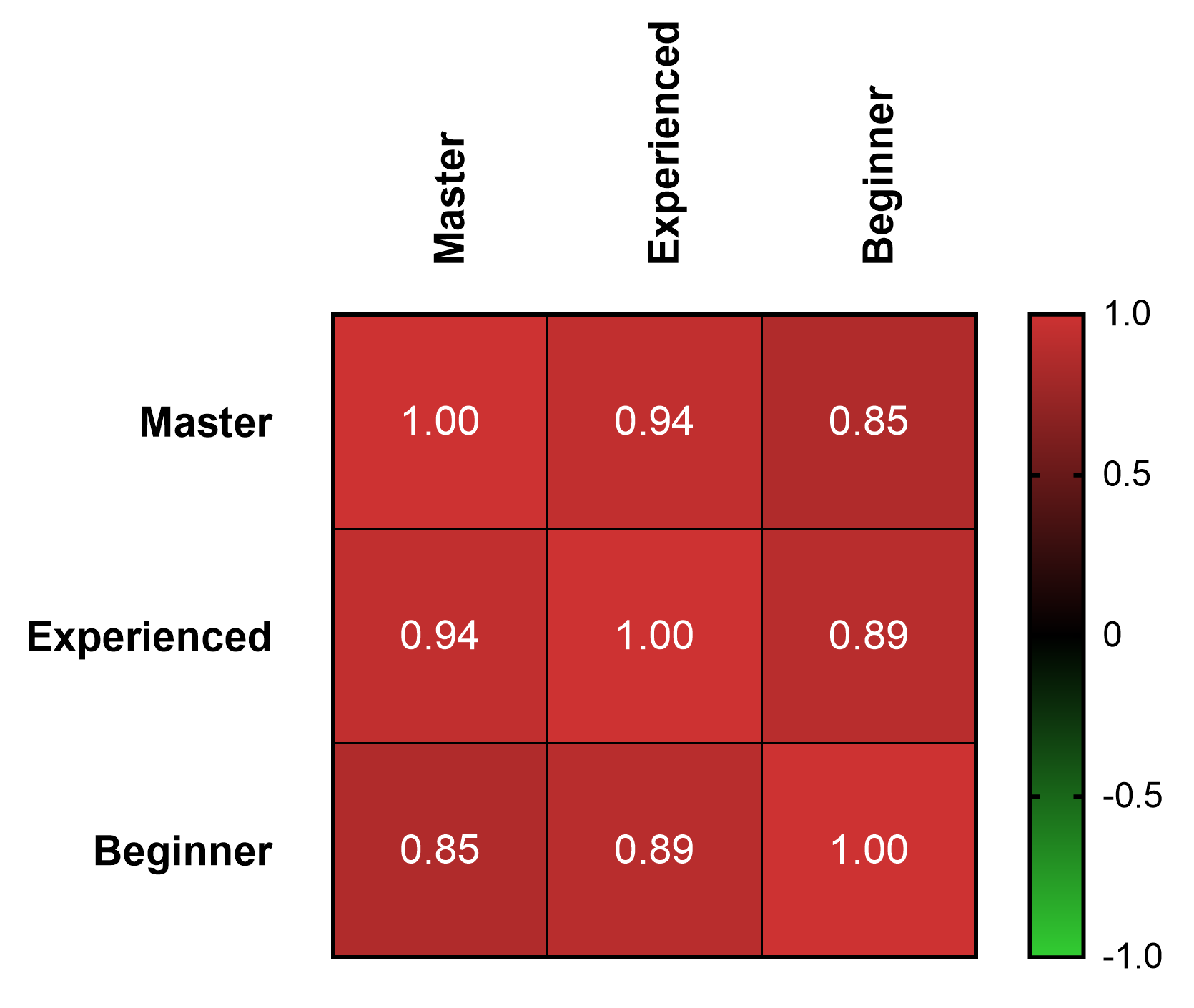

Supplement: Extended Data Figure 3-1 — Inter-rater variability analysis between human scorers of varying experience levels. In short, 20 behavioral videos (counterbalanced for IST/DST and objects/odors) were scored for rat-stimulus interaction by three independent scorers of differing experience levels (master, experienced, beginner). We found a strong correlation between scorers of all experience levels, but a comparatively weaker correlation between experienced and beginner scorers. Download Figure 3-1, TIF file. [file enu-eN-NWR-0115-23-s06.tif]

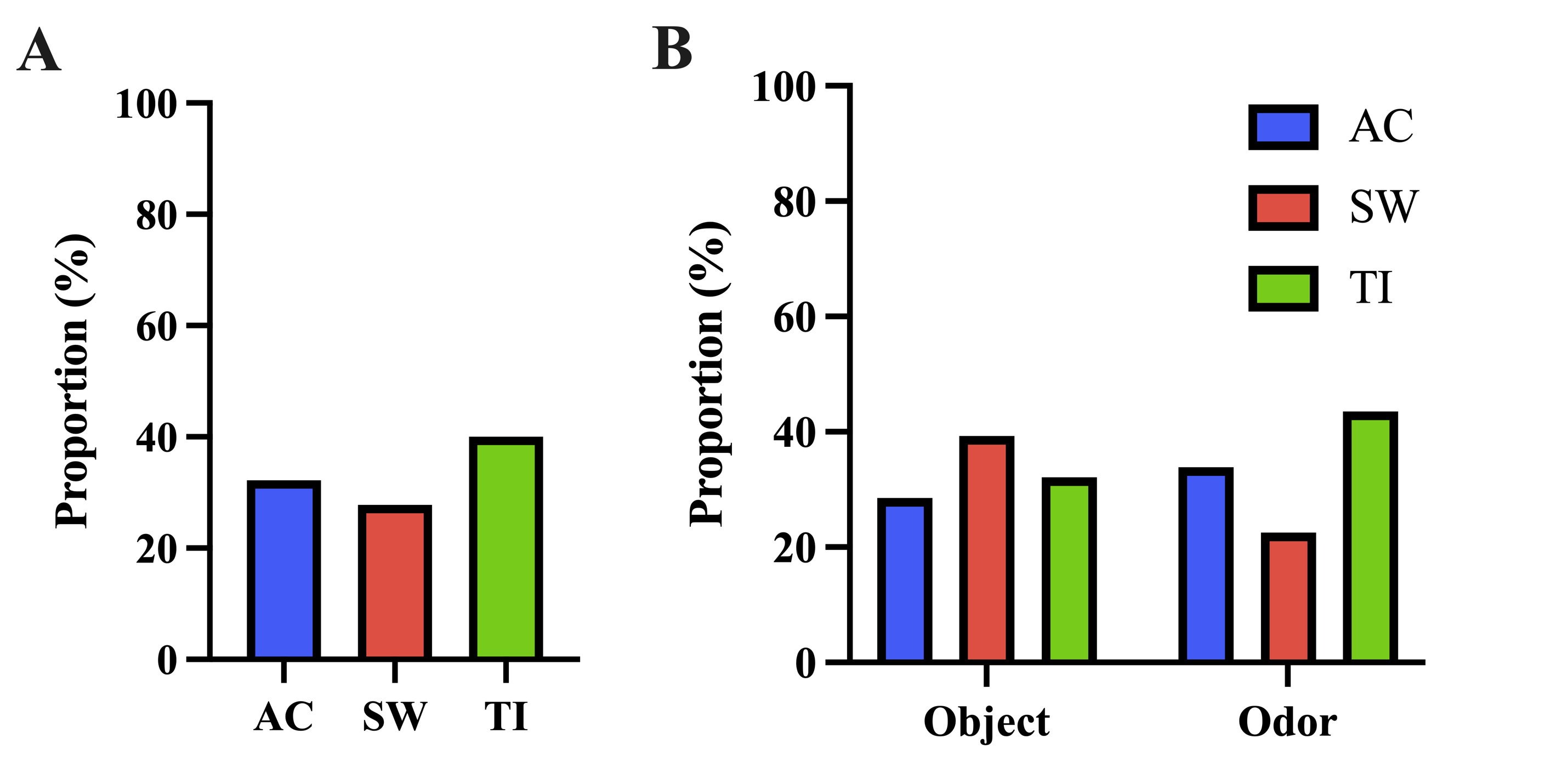

Supplement: Extended Data Figure 3-2 — Proportion of excluded videos from verification ranks 4 and 5 as described in Figure 3C,D. The proportion of videos excluded did not differ significantly when grouped by treatment (A) or stimuli type (B). Download Figure 3-2, TIF file. [file enu-eN-NWR-0115-23-s07.tif]
